# Machine Learning for Human-Autonomy Teaming in Surgical Skill Assessment: Scoping Review

**DOI:** 10.2196/94109

**Published:** 2026-10-02

**Authors:** Kamal Barati, Michael S Ramirez Campos, Thomas E Doyle

**Affiliations:** 1 Department of Electrical and Computer Engineering McMaster University Hamilton, ON Canada; 2 School of Biomedical Engineering McMaster University Hamilton, ON Canada

**Keywords:** deep learning, human-autonomy teaming, machine learning, multimodal data, surgical skill assessment, technology readiness level

## Abstract

**Background:**

Human-autonomy teaming (HAT) has the potential to reshape surgical practice by fostering true partnership between surgeons and intelligent systems. To achieve this, AI must move beyond static scoring to provide adaptive, real-time guidance based on reliable skill assessment.

**Objective:**

This scoping review aims to map the current landscape of machine learning methods for surgical skill assessment and evaluate their technical readiness for integration into functional HAT systems.

**Methods:**

Following PRISMA-ScR (Preferred Reporting Items for Systematic Reviews and Meta-Analyses extension for Scoping Reviews) guidelines, we conducted a systematic search across 3 major scientific databases (PubMed, IEEE Xplore, and Web of Science). We identified and analyzed 92 peer-reviewed studies published between 2019 and 2025. The review focused on data modalities (kinematics, video, and biosignals), model architectures, and validation environments. To assess translational maturity, each study was evaluated using the NASA technology readiness level (TRL) scale and mapped to the Yang et al levels of autonomy for surgical robotics, alongside 6 additional dimensions: real-time capability, interpretability, adaptivity, validation environment, external validation, and clinical integration.

**Results:**

Our analysis of the 92 included studies reveals a dominant shift toward multimodal data integration and deep learning architectures. While high performance is frequently reported on benchmark datasets, significant barriers to HAT integration persist. The TRL analysis shows that 77.2% (71/92) of studies remain at TRL 3 (proof-of-concept), with only 2.2% (2/92) reaching TRL 5 or above. Furthermore, 96.7% (89/92) of studies operate at Yang autonomy level 0, providing no autonomous functionality beyond classification. We identified that 91.3% (84/92) of models are static, with no user-specific adaptation, 38% (35/92) lack any external validation, and validation practices remain inconsistent across the field.

**Conclusions:**

Current AI techniques provide a robust foundation for objective skill assessment, but they are not yet ready for autonomous teaming. Future development must prioritize model robustness, interpretability, and seamless integration into clinical environments to transition from stand-alone assessment tools to effective surgical teammates.

**Trial Registration:**

OSF Registries 10.17605/OSF.IO/PQWS5; https://osf.io/pqws5

## Introduction

Assessing surgical skill is essential for both training and ensuring safe and effective procedures. Traditionally, this has been done through expert observation and standardized rating tools such as the Objective Structured Assessment of Technical Skills (OSATS) [1] or the Global Rating Scale (GRS). Although widely used, these methods are time-consuming, subjective, and not always consistent between raters. Advances in surgical simulation platforms, robotic systems, and sensing technologies—such as motion tracking, video, and biosignal acquisition—offer new opportunities for scalable, objective, and data-driven assessment. In this context, machine learning (ML) has emerged as a transformative tool [2,3] capable of analyzing multimodal data [4,5] to evaluate surgical performance with high accuracy and consistency. Surgical competence encompasses both technical skills (eg, instrument handling and tissue manipulation) and nontechnical skills (eg, communication, decision-making, and situational awareness) [6-8]. This review focuses specifically on the assessment of technical skills using ML methods.

Researchers have explored various ML models to analyze surgical performance using data sources such as tool trajectories, surgical video, force signals, and physiological measurements. These models are often trained to classify levels of expertise, recognize gestures or procedural phases, or evaluate metrics such as efficiency, motion, and safety. For instance, convolutional neural networks (CNNs) and recurrent models such as long short-term memory (LSTM) networks have been applied to video and motion data, while more recent work incorporates transformers for sequence modeling [4,5,9,10]. This computational approach enables consistent and scalable evaluation of surgical skill across settings.

Much of the foundational work in ML for surgical skill assessment has relied on benchmark datasets such as JIGSAWS (Johns Hopkins University and Intuitive Surgical Inc Gesture and Skill Assessment Working Set), which includes synchronized kinematic and video data of simulated robotic tasks performed by participants with varying levels of expertise [11-13]. While this dataset has enabled reproducible research and model benchmarking, it is limited by its small sample size, controlled simulation environment, and narrow scope of tasks. As a result, models trained on such data may struggle to generalize to real-world surgical settings, which are inherently more complex and variable.

To address these limitations, recent studies have shifted toward using clinical data, including videos of real procedures, cadaver models, and live intraoperative recordings [3,5,14]. These sources offer greater realism and diversity, enabling ML models to capture variability in anatomy, surgeon technique, and surgical context. In addition, advances in sensor technologies have made it possible to integrate physiological signals (eg, electroencephalography and electrocardiography), instrument motion, and force data into multimodal learning frameworks [2,10]. This shift opens the door to not only more accurate post hoc evaluations but also the development of real-time, adaptive systems that can assist, guide, or learn alongside human operators [2,3].

Human-autonomy teaming (HAT) represents a paradigm shift from traditional human-machine interaction toward collaborative partnerships between humans and intelligent systems. Unlike conventional automation, which operates in isolation or under strict human control, HAT involves dynamic cooperation—where decision-making, situational awareness, and task execution are shared across the human and autonomous agent [15,16]. In surgical settings, this could involve AI systems that analyze procedural context in real time, adapt their behavior based on a surgeon’s skill level, or provide tailored feedback to enhance performance and learning. As such, HAT is not just a technological evolution but a transformation in how expertise, responsibility, and adaptability are distributed in high-stakes clinical environments.

This review uses an established conceptual framework, adopting the levels of autonomy for surgical robotics proposed by Yang et al [6], which defines 6 stages ranging from level 0 (no autonomy; pure teleoperation) through level 1 (robot assistance, such as virtual fixtures or tremor filtering), level 2 (task autonomy, where the system executes specific subtasks under continuous supervision), level 3 (conditional autonomy, with the robot performing extended sequences while the surgeon monitors and intervenes as needed), to levels 4 and 5 (high and full autonomy, requiring minimal or no human involvement). This taxonomy provides a structured lens through which the technical maturity of current ML-based skill assessment methods can be evaluated. Most studies reviewed in this work operate at or below level 1—providing post hoc classification or passive feedback—while the transition toward HAT demands capabilities aligned with levels 2 and 3, where the system must interpret surgical context in real time, adapt to individual performance, and actively support decision-making within the procedural workflow. By mapping the reviewed literature against this framework, we aim to identify not only what current models achieve but also how far the field remains from enabling genuine human-autonomy collaboration in surgery.

While HAT holds great promise for enhancing surgical decision-making and skill development, its implementation presents several open challenges. Effective teaming requires AI systems that can interpret the surgical context in real time and provide feedback tailored to the surgeon’s level of expertise—whether novice, intermediate, or expert. However, developing such adaptive systems is complex. Many current models are trained on limited datasets that fail to capture the variability of real-world surgical environments, reducing their generalizability and robustness [12,14]. Additionally, the lack of transparency in deep learning systems can erode clinical trust, particularly when decisions affect patient outcomes. Real-time integration into the operating room also raises concerns around system latency, interoperability, and regulatory approval. Finally, ethical considerations such as accountability, autonomy, and the transparency of shared decision-making remain underexplored. These limitations highlight the need for a comprehensive synthesis of current research, particularly regarding how HAT systems can adapt to different users and safely support surgical workflows in real time.

To guide this review, we posed the following research questions (RQs):

1. How can HAT models be adapted to assist in real-time surgical decision-making and skill development, focusing on personalized guidance and feedback?
2. What ML methods exist to classify surgical movements and assess surgeon proficiency, and how can multimodal data (eg, video, kinematic, and force sensors) enhance this classification for real-time feedback?
3. What are the benchmarks for evaluating the accuracy and effectiveness of AI-driven movement-based skill assessments, and how do they compare with expert evaluations?

This review presents a systematic account of the current landscape of ML techniques for surgical skill assessment, with particular attention to (1) model design, (2) data modality integration, and (3) readiness for HAT. We synthesize findings across 92 peer-reviewed studies and identify gaps in validation practices, generalizability, and clinical integration. These insights are critical as the field moves toward deploying AI systems that not only evaluate performance but also collaborate with human surgeons in real-time, ultimately shaping the future of surgical education and patient care.

## Methods

### Overview

This scoping review follows the methodological framework proposed by Arksey and O’Malley [17], as refined by Levac et al [18], and is reported following the PRISMA-ScR (Preferred Reporting Items for Systematic Reviews and Meta-Analyses extension for Scoping Reviews) guidelines (Multimedia Appendix 1) [19,20]. As outlined, this review presents a systematic mapping of existing AI applications in surgical skill assessment, with a particular emphasis on HAT, real-time guidance, and multimodal learning approaches. The protocol for this review was registered on the Open Science Framework prior to data extraction [21]. Two deviations from the registered protocol occurred: (1) 3 additional records were identified through manual searching of reference lists, supplementing the database search described in the protocol; and (2) a technology readiness level (TRL) assessment framework and supplementary translational readiness dimensions (Yang autonomy level, real-time capability, interpretability, adaptivity, validation environment, external validation, and clinical integration) were added during revision in response to peer review feedback to strengthen the analytical evaluation of included studies. Neither deviation affected the screening criteria or the final corpus of included studies.

To ensure consistent application, the following operational definitions guided the coding. Real-time capability was classified as “real-time” when inference was demonstrated during live task execution, “near-real-time” when the authors reported fast inference speeds but did not demonstrate integration into a live workflow, and “offline” when analysis was performed retrospectively. Interpretability was coded as “built-in” for architectures with inherently transparent components (eg, decision trees and rule-based systems), “post hoc” when dedicated explanation methods such as Shapley additive explanations (SHAP) or gradient-weighted class activation mapping (Grad-CAM) were applied, and “none” otherwise; attention mechanisms and feature importance rankings were coded as “built-in” where the study explicitly used them to explain model decisions, but this classification is acknowledged as a simplification. External validation was subcategorized as cross-task (different tasks within the same dataset), cross-dataset (a different dataset entirely), and cross-site (data from a different institution), reflecting increasing levels of generalizability rigor. Clinical integration was coded as “detailed” when the study included substantive discussion of deployment considerations, workflow integration, or regulatory aspects, and “brief” when clinical relevance was mentioned without elaboration.

### Search Strategy

A structured search was conducted in Web of Science, IEEE Xplore, and PubMed to identify peer-reviewed articles published between January 2019 and July 2025. The search targeted studies on surgical skill assessment and assisted decision-making using ML or related AI approaches. Search terms included combinations of the following: *human-autonomy teaming*, *surgical skill assessment*, *surgical gesture recognition*, *robotic surgery*, *machine learning*, *deep learning*, *multimodal data*, and *real-time feedback*. Boolean operators (AND, OR) were used to structure the queries and adapt them to each database’s syntax (Multimedia Appendix 2).

An example search string used for Web of Science was:

ALL=((“surgical movement classification” OR “surgical skill assessment” OR “surgical training AI” OR “gesture recognition surgery” OR “kinematic analysis surgery” OR “surgical education”) OR (“AI-assisted decision making” OR “human-autonomy teaming in surgery” OR “real-time surgical feedback” OR “personalized surgical guidance”)) AND (ALL=(“Artificial Intelligence” OR “Machine Learning” OR “Deep Learning”))

Search results were imported into Covidence, and duplicate entries were removed prior to screening.

### Inclusion and Exclusion Criteria

Textbox 1 defines the inclusion and exclusion criteria to select studies for full-text review.

**Inclusion and exclusion criteria for studies.**

**Inclusion criteria**

- Studies focused on surgical skill assessment, AI-based classification of surgical movements, human-autonomy teaming, or decision-making for surgical tasks.
- Use of machine learning techniques (eg, convolutional neural networks, recurrent neural networks, transformers, or graph convolutional networks) or statistical approaches such as linear or logistic regression.
- Studies addressing real-time or near-real-time feedback, movement classification, or benchmarks related to AI-driven skill assessment.
- Populations involving surgical trainees (medical students, residents, and fellows), experienced surgeons, or robotic systems in surgical skill development contexts.
- Studies using kinematic, video, electroencephalography, or electrocardiogram data for analysis.
- Original research articles (excluding reviews or summaries).
- Published within the past 7 years (2019-2025).

**Exclusion criteria**

- Studies unrelated to surgical skill assessment, AI methods, or decision-making.
- Articles that do not focus on AI-driven movement classification, decision support, or benchmarking of skills.
- Studies that do not use any type of recorded or measured data.
- Preprints, review articles, or non–peer-reviewed content.
- Studies lacking experimental validation or empirical results.
- Articles focusing solely on ethics, philosophy, or conceptual discussions without technical or methodological contributions.

### Screening and Selection

A total of 1139 records were identified, comprising 391 from PubMed, 193 from IEEE Xplore, 552 from Web of Science, and 3 additional records identified through manual searching of reference lists and related literature. After duplicate removal, a total of 506 papers were screened based on title and abstract to remove clearly irrelevant studies. This left 130 articles for full-text review. After applying the inclusion/exclusion criteria, 92 papers were selected for final synthesis. Screening and selection were independently performed by 2 reviewers (the first author and a collaborator), with disagreements resolved through discussion.

### Data Extraction and Analysis

For each included study, information was extracted regarding the publication, ML methods used, type of surgical task, data modalities (eg, kinematics, video, and electroencephalography), population (eg, novices, experts, and robotic systems), model outputs, and how performance was evaluated.

Studies were then grouped into thematic clusters based on learning method (eg, deep learning vs rule-based), data modality (eg, video-only vs multimodal), and clinical focus (eg, simulation tasks vs real surgeries). This allowed us to compare trends across time, methods, and application domains.

### Analytical Framework for Technology Readiness

To systematically evaluate the translational maturity of the reviewed studies, we adopted the NASA TRL scale [22], a widely used framework for assessing the maturity of emerging technologies from basic research (TRL 1) through proven operational deployment (TRL 9). We adapted the 9-level scale to the surgical ML context as follows: TRL 1-2 corresponds to basic algorithmic research and concept formulation; TRL 3 to proof-of-concept models tested on limited or benchmark data (eg, JIGSAWS); TRL 4 to models validated in laboratory or simulation environments with standard metrics; TRL 5 to models tested on clinical or cross-institutional data; TRL 6-7 to prototype systems demonstrated in simulated or real operating room environments; and TRL 8-9 to clinically validated, regulatory-ready, or routinely deployed systems.

In addition to TRL, each study was assessed along 6 translational dimensions: real-time capability (offline, near-real-time, or real-time inference), interpretability (none, post hoc, or built-in), adaptivity to individual users (none, skill-stratified, or adaptive), validation environment (simulation, laboratory/cadaver, clinical, or mixed), external validation (none, cross-task, cross-dataset, or cross-site), and clinical integration discussed (none, brief, or detailed). These dimensions, together with the TRL and the Yang et al [6] autonomy levels introduced in the Introduction, provide a structured lens for evaluating how far current approaches have progressed toward functional HAT integration. It should be noted that both the TRL and Yang frameworks were applied post hoc as analytical lenses rather than as instruments originally designed for evaluating ML classification studies. In particular, Yang level 0 was used in this review to indicate the absence of any autonomous system functionality, rather than its original meaning of strict teleoperation, since the majority of reviewed studies evaluate algorithms in isolation rather than within an integrated robotic system. These adaptations represent a pragmatic approximation, and their limitations should be considered when interpreting the results.

The TRL and translational dimension assessments were conducted in 2 stages. First, TRLs were assigned independently by 2 reviewers (KB and MSRC) for all 92 studies. Initial percent agreement on TRL assignment was 67.4% (62/92 studies). Disagreements occurred predominantly at the TRL 3-4 boundary, reflecting differing interpretations of whether laboratory-based validation with human participants constituted component validation (TRL 4) or remained at proof-of-concept level (TRL 3). All 30 disagreements were resolved through discussion, during which explicit decision rules were refined: studies validated exclusively on benchmark or preexisting datasets were assigned TRL 3, while those integrating multiple components (hardware, software, and participants) in a laboratory setting were assigned TRL 4. Second, the remaining translational dimensions (Yang autonomy level, real-time capability, interpretability, adaptivity, validation environment, external validation, and clinical integration) were coded by the first author (KB) using the operational definitions described above and verified by the second reviewer (MSRC), with discrepancies resolved through discussion. The final consensus ratings are reported throughout this review.

### Ethics Approval

This study is a scoping review of previously published research and did not involve the collection of new data from human participants. Therefore, it did not require ethical approval or informed consent according to institutional and national guidelines. All studies included in this review were assumed to have obtained appropriate ethical approval and to have adhered to relevant regulations.

## Results

### Overview

The PRISMA (Preferred Reporting Items for Systematic Reviews and Meta-Analyses) flow diagram outlining the study selection process is presented in Figure 1. This review includes 92 studies published between 2019 and early 2025. Most studies were published after 2020, reflecting a growing interest in applying ML to surgical skill assessment, especially in simulation and robotic-assisted surgery contexts.

**Figure 1:**
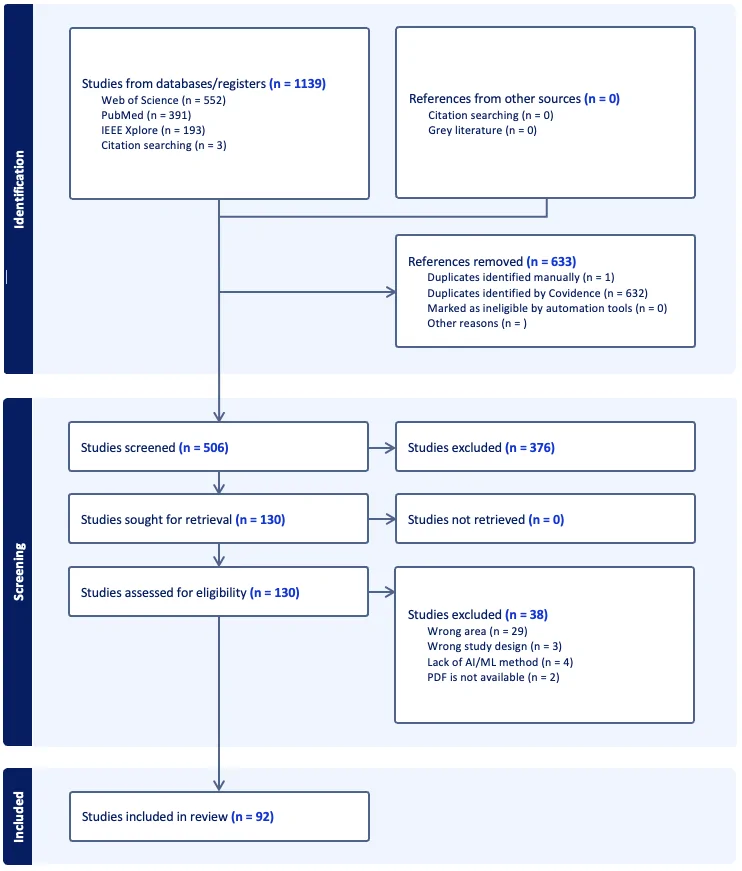
PRISMA (Preferred Reporting Items for Systematic Reviews and Meta-Analyses) flow diagram outlining the selection process for included studies.

Table 1 summarizes the distribution of translational readiness characteristics across the 92 included studies. The full per-study assessment is provided in Table S1 in Multimedia Appendix 3.

**Table 1:** Distribution of translational readiness characteristics across included studies (n=92).

Dimension and category		Studies, n (%)
**TRL^a^ level**		
	Level 3	71 (77.2)
	Level 4	19 (20.7)
	Level 5	1 (1.1)
	Level 7	1 (1.1)
**Yang autonomy level**		
	Level 0	89 (96.7)
	Level 1	3 (3.3)
**Real-time capability**		
	Offline	53 (57.6)
	Near-real-time	16 (17.4)
	Real-time	23 (25.0)
**Interpretability**		
	None	21 (22.8)
	Post hoc	5 (5.4)
	Built-in	66 (71.7)
**Adaptivity**		
	None	84 (91.3)
	Skill-stratified	2 (2.2)
	Adaptive	6 (6.5)
**Validation environment**		
	Simulation only	14 (15.2)
	Laboratory/cadaver	39 (42.4)
	Clinical	26 (28.3)
	Mixed	13 (14.1)
**External validation**		
	No	35 (38)
	Cross-task	24 (26.1)
	Cross-dataset	19 (20.7)
	Cross-site	14 (15.2)
**Clinical integration**		
	Brief	78 (84.8)
	Detailed	14 (15.2)

^a^TRL: technology readiness level.

The studies covered a diverse range of surgical tasks and environments (Table 2). This review found an even split between simulation-based and clinical-focused research. Specifically, 46 of the papers used simulation-based setups, such as benchmark datasets (eg, JIGSAWS or Cholecystectomy 80 [Cholec80]), virtual reality (VR) simulators (eg, NeuroVR, ANGIO Mentor, or Fundamentals of Robotic Surgery Dome), bench-top models, or simple box trainers. The remaining 46 papers focused on clinical or semiclinical data, including real patient procedures, wet laboratory training on animal models, cadaver laboratories, or in vivo videos recorded in operating rooms. This balanced approach demonstrates the field’s maturity in transitioning from controlled laboratory validation to real-world clinical evaluation. A few studies notably focused on low-cost custom-built platforms such as the Advanced Robotics and Automated System-Farabi platform for capsulorhexis training.

**Table 2:** Distribution of included studies by application focus and data modality.

		Studies, n
**Application focus**		
	Gesture recognition or segmentation	17
	Skill classification	56
	Tool tracking and motion analysis	29
	Real-time feedback or assessment systems	18
**Data modality**		
	Kinematic data	18
	Video data (RGB, optical flow, etc)	58
	Physiological data (EEG^a^, fNIRS^b^, etc)	3
	Force data	1
	Multimodal approaches (2+modalities fused)	12

^a^EEG: electroencephalography.

^b^fNIRS: functional near-infrared spectroscopy.

The data modalities varied significantly (Table 2): video was the primary input for 58 (63%) papers; 18 (19.6%) used kinematic data, often from the JIGSAWS [12] or da Vinci Research Kit platforms; a smaller group of 12 (13%) used multimodal data combining inputs such as video, kinematics, pose estimation, or tool trajectory features [23]; 3 looked at physiological signals such as electroencephalography or functional near-infrared spectroscopy (n=3, 3.3%); and 1 focused on force signals (n=1, 1.1%).

The study populations also varied across the literature. The vast majority of studies, 68 (73.9%) papers, involved mixed-skill groups that included a combination of trainees, residents, fellows, and expert surgeons [2,24]. Another 14 (15.2%) papers did not focus on a specific clinical cohort, instead using preexisting datasets (eg, JIGSAWS) or concentrating on the development of the AI system itself [25,26]. A smaller number of studies focused exclusively on a single group: 6 (6.5%) papers studied only trainees such as medical students [27], while 4 (4.3%) papers analyzed data from only expert surgeons [28].

The data sources used for analysis were highly diverse. The most common approach, found in 29 (31.5%) studies, involved simulation platforms other than the JIGSAWS benchmark; these included high-fidelity VR trainers for neurosurgery and robotic tasks, as well as physical box trainers for fundamental surgical skills [4,29]. Data from real clinical or in vivo procedures on patients or animal models formed the basis for 28 (30.4%) studies, which analyzed a wide range of surgeries such as live robotic prostatectomies, cataract surgery, and cholecystectomies [5,30]. Another 18 (19.6%) studies developed or used custom or other public datasets, including newly annotated video collections (eg, Cholec80) and unique experimental setups [14,31]. Finally, the standardized JIGSAWS benchmark dataset was the primary data source for 17 (18.5%) studies, which leveraged its kinematic and video data for tasks such as suturing and knot tying [9,32].

Overall, these 92 studies cover a wide cross-section of methods, datasets, and goals, which are presented in Tables 3-6. There has been a clear shift toward integrating different data types, moving beyond offline analysis, and thinking more about how AI systems might work alongside people—rather than evaluate them. These directions are especially important when we start to think about AI as an active teammate, not just a passive tool.

**Table 3:** Summary of included studies on skill classification and regression.

Study	Year	Data modality	Data source	Sample size	Assessment target/outcome	ML^a^ method	Data availability
[33]	2025	Video	Simulator	N/A^b^	Laparoscopic skill validation	YOLOv4^c^	Private
[34]	2025	Video	Simulator	N/A	Technical skill classification	Vision Transformer	Private
[35]	2025	Text (free-text comments)	Simulator (Vertex Pursuit)	600 data points (24 participants)	Skill level classification (novice, intermediate, and expert)	LLM^d^ (Claude 3 Opus, GPT-4, Gemini Pro); BERT^e^; SVM^f^; KNN^g^; RF^h^; XGBoost^i^; MLP^j^	Private
[36]	2025	Video	Clinical (colorectal)	N/A	Skill assessment	CNN^k^ (EfficientNetB7)	Private
[37]	2025	Motion (rotation)	Simulator	N/A	Suturing skill assessment (hand rotation)	Functional data analysis	Private
[38]	2025	Video (tool motion)	Simulator (EA OJT^l^ Model)	45 videos	Skill level classification (good vs poor)	YOLOv3; CNN (4-layer)	Private
[39]	2025	Video	Clinical (cataract)	>1000 videos	OSACSS^m^ performance ratings	YOLACT^n^	Private
[10]	2024	Kinematics; force	VR^o^/AR^p^ simulator	27 participants	Expertise classification	ANN^q^; transfer learning	Private
[40]	2024	Video (surgestures)	Clinical (laparoscopic cholecystectomy)	75 videos (33 surgeons)	mGOALS^r^ skill classification (competent vs incompetent)	SVM; LR^s^; RF; GBDT^t^; AdaBoost	Private
[41]	2024	Video	EndoVis SkillNet	N/A	Skill classification	Video Transformer (multitask)	Public
[42]	2024	Video	Suture simulator	314 videos	GRS^u^ score classification (novice, intermediate, and proficient)	Video Swin Transformer; I3D^v^	Public
[43]	2024	Video	Clinical (laparoscopic distal gastrectomy)	256 videos (training); 180 videos (testing)	ESSQS^w^ skill score (phase duration and AICS^x^)	CNN (EfficientNetB7)	Private
[44]	2024	Video	Clinical (laparoscopic colorectal)	1572 videos	ESSQS skill score regression	EfficientNetB7; regression	Private
[24]	2023	Video (motion)	Laparoscopic trainer (LMICs^y^)	74 videos (47 trainees)	OSATS^z^ score prediction	KNN; YOLOv5	Private
[45]	2024	EEG^aa^; eye-tracking	Clinical (porcine model)	43 surgeries (11 surgeons)	GEARS^ab^ skill level classification	RF; XGBoost; LR; gradient boosting	Private
[46]	2024	Force	Simulator	N/A	Skill assessment (Smoothness of Force)	Time/frequency analysis	Private
[47]	2024	Motion (EM^ac^ sensors)	SutureCoach Simulator	97 participants	Expertise level classification (novice, intermediate, and expert)	Statistical metrics	Private
[48]	2024	Kinematics	dVRK^ad^	N/A	Bimanual coordination assessment	Information theory; DTW^ae^	Private
[30]	2024	Video	Clinical (cataract surgery)	197 videos (12 surgeons)	Skill level classification (expert vs novice)	2D CNN-LSTM^af^; 3D CNN	Private
[49]	2024	Kinematics	JIGSAWS^ag^; Wolf dataset	8 (JIGSAWS); 32 (Wolf)	GRS item score prediction (GOALS^ah^/OSATS)	Multiheaded MLP	Public
[9]	2024	Kinematics (as images)	JIGSAWS	8 participants	Skill level classification	Vision Transformer; CWT^ai^	Public
[50]	2023	Video	JIGSAWS	8 participants	GRS score prediction (relative to expert)	Transformer; TCN^aj^; ResNet^ak^-18	Public
[51]	2023	Video	Clinical (laparoscopic sigmoidectomy)	120 videos	ESSQS score correlation (AICS)	CNN (Xception)	Private
[52]	2023	Kinematics	JIGSAWS	8 participants	Skill level classification (novice, intermediate, and expert)	Dempster-Shafer theory; SVM; XGBoost	Public
[2]	2023	EEG	VR simulator (NeuroVR)	21 participants	Expertise level classification (skilled vs less-skilled)	ANN; SVM; LDA^al^; RF; KNN; LR; naive Bayes	Private
[53]	2023	Video (tool motion)	JIGSAWS	24 videos (8 participants)	Skill level classification (2-class and 3-class)	ResNet; KCF^am^ tracking	Public
[54]	2023	Simulator metrics	ANGIO Mentor Simulator	22 participants	Modified Reznick scale score prediction	Feedforward neural network	Private
[16]	2023	Kinematics	JIGSAWS; VR simulator	8 (JIGSAWS); 16 (VR)	Skill level classification (novice vs expert)	CNN; Bi-LSTM^an^; MC^ao^ Dropout	Public
[55]	2023	Video (tool motion)	JIGSAWS; FLS^ap^ trainer	8 (JIGSAWS); 33 (FLS)	Pass/fail classification	Res-CNN^aq^; DAE^ar^; Mask R-CNN^as^; CAM^at^	Public
[56]	2022	Motion capture	Wet Lab box trainer	89 sessions (70 participants)	GOALS score prediction	SVR^au^; PCA^av^; Ridge; PLS^aw^ Regression	Private
[57]	2022	Video (hand motion)	Laparoscopic simulator	9 participants	Hand movement performance assessment	Fuzzy logic; SSD^ax^ ResNet50	Public
[58]	2022	Video (tool motion)	Cholec80	80 videos (13 surgeons)	GOALS efficiency score classification	Transformer; random forest; YOLOv5	Public
[59]	2022	Kinematics; image	Knot-tying simulator	360 trials (72 trainees)	OSATS score prediction (4 domains)	ResNet; Bi-LSTM	Public
[60]	2022	VR simulators	VR simulators	254 sessions	GEARS score classification (novice, intermediate, and expert)	DNN^ay^ (Google AutoML)	Private
[27]	2022	fNIRS^az^	FLS Trainer	474 trials (7 students)	Pass/fail classification (FLS task)	Dilated causal CNN (WaveNet-style)	Private
[61]	2022	Motion; force	Phantom; clinical	12 surgeons	Skill level classification (expert vs novice)	RF; XGBoost; SHAP^ba^	Private
[62]	2022	Kinematics (1D)	Clinical (porcine model)	100 trials (10 surgeons)	Intra- and intersubject skill similarity	Dynamic time warping	Private
[4]	2021	Kinematics; force	VR Simulator (Sim-Ortho)	23 participants	Expertise classification	ANN (multilayer perceptron)	Private
[63]	2021	Kinematics	JIGSAWS	33 trials (7 surgeons)	Skill level classification (novice, intermediate, and expert)	KNN; PCA; Relief	Public
[64]	2021	Video (optical flow)	JIGSAWS	8 participants	Skill level classification (novice vs expert)	ResNet; CNN; LSTM; ConvAuto	Public
[65]	2021	Video (tool motion)	Clinical (cholecystectomy)	949 clips	Likert scale skill score prediction (1-5)	Linear regression; ResNet50-FPN^bb^	Private
[66]	2021	Video; kinematics	JIGSAWS; Clinical (laparoscopic gastrectomy)	206 videos (JIGSAWS); 20 videos (clinical)	GRS score prediction (modified OSATS)	TCN; ResNet-101; MS-TCN^bc^; MLP; contrastive learning	Public, Private
[67]	2021	Video	JIGSAWS	8 participants	GRS score classification (expert vs novice)	SVM; BoF^bd^ (STIP^be^, iDT^bf^)	Public
[12]	2021	Video	JIGSAWS	8 participants	Skill level classification (novice, intermediate, and expert)	ResNet50; FFT^bg^; 1D CNN	Public
[68]	2021	Sensor (force, time)	FRS^bh^ Dome Simulator	27 performances	FRS performance score (1-3)	ANFIS^bi^; fuzzy logic	Private
[32]	2020	Kinematics	JIGSAWS	8 participants	Skill level classification	CNN; LSTM; PCA; DFT^bj^; DCT^bk^; Autoencoder; SVM	Public
[69]	2020	Video	JIGSAWS	8 participants	OSATS score prediction	I3D; TSN^bl^ (multitask)	Public
[70]	2020	Video (tool motion)	Clinical (robotic thyroidectomy); Simulator (BABA Model)	54 video segments	OSATS/GEARS skill classification (novice, skilled, and expert)	Mask R-CNN; Deep SORT^bm^; SVM; random forest	Private
[71]	2020	Kinematics	JIGSAWS	8 participants	GRS score classification (expert vs novice)	Kernel SVM	Public
[72]	2019	Kinematics	JIGSAWS	8 participants	OSATS score prediction	FCN^bn^; CAM	Public
[73]	2019	Video; optical flow	JIGSAWS	8 participants	Skill level classification (novice, intermediate, and expert)	3D CNN (I3D); TSN	Public
[74]	2019	Video (tool motion)	UVA^bo^ Dataset	12 surgeons	Skill level classification (novice vs expert)	HMM^bp^; SVM; LDA	Public
[75]	2019	Video (tool motion, optical flow)	Clinical (cataract surgery)	99 videos	ICO-OSCAR^bq^ score prediction (expert vs nonexpert)	TCN	Private
[76]	2019	EMG^br^; motion	Myo Armband (box trainer)	99 knots (28 participants)	OSATS score prediction	Neural network; decision trees	Private
[77]	2019	Video (color, semantic)	Clinical (laparoscopic gastrectomy)	57 videos (1 surgeon)	OSATS/COF^bs^ score prediction	ResNet-101; regression; rank loss	Private

^a^ML: machine learning.

^b^N/A: not available.

^c^YOLOv4: You Only Look Once version 4.

^d^LLM: large language model.

^e^BERT: Bidirectional Encoder Representations from Transformers.

^f^SVM: support vector machine.

^g^KNN: k-nearest neighbors.

^h^RF: random forest.

^i^XGBoost: extreme gradient boosting.

^j^MLP: multilayer perceptron.

^k^CNN: convolutional neural network.

^l^EA OJT: esophageal atresia off-the-job training.

^m^OSACSS: Objective Structured Assessment of Cataract Surgical Skill.

^n^YOLACT: You Only Look at Coefficients.

^o^VR: virtual reality.

^p^AR: augmented reality.

^q^ANN: artificial neural network.

^r^mGOALS: Modified Global Objective Assessment of Laparoscopic Skills.

^s^LR: logistic regression.

^t^GBDT: gradient boosted decision trees.

^u^GRS: Global Rating Scale.

^v^I3D: Inflated 3D ConvNet.

^w^ESSQS: Endoscopic Surgical Skill Qualification System.

^x^AICS: Artificial Intelligence Confidence Score.

^y^LMIC: low-and-middle-income country.

^z^OSATS: Objective Structured Assessment of Technical Skills.

^aa^EEG: electroencephalography.

^ab^GEARS: Global Evaluative Assessment of Robotic Skills.

^ac^EM: electromagnetic.

^ad^dVRK: da Vinci Research Kit.

^ae^DTW: dynamic time warping.

^af^LSTM: long short-term memory.

^ag^JIGSAWS: Johns Hopkins University and Intuitive Surgical Inc Gesture and Skill Assessment Working Set.

^ah^GOALS: Global Objective Assessment of Laparoscopic Skills.

^ai^CWT: continuous wavelet transform.

^aj^TCN: temporal convolutional network.

^ak^ResNet: residual network.

^al^LDA: linear discriminant analysis.

^am^ANGIO: angiotherapy.

^an^Bi-LSTM: bidirectional long short-term memory.

^ao^MC: Monte Carlo.

^ap^FLS: Fundamentals of Laparoscopic Surgery.

^aq^Res-CNN: residual convolutional neural network.

^ar^DAE: denoising autoencoder.

^as^Mask R-CNN: mask region-based convolutional neural network.

^at^CAM: class activation mapping.

^au^SVR: support vector regression.

^av^PCA: principal component analysis.

^aw^PLS: partial least squares.

^ax^SSD: single shot detector.

^ay^DNN: deep neural network.

^az^fNIRS: functional near-infrared spectroscopy.

^ba^SHAP: Shapley additive explanations.

^bb^FPN: feature pyramid network.

^bc^MS-TCN: multistage temporal convolutional networks.

^bd^BoF: bag of features.

^be^STIP: spatio-temporal interest points.

^bf^iDT: improved dense trajectories.

^bg^FFT: fast Fourier transform.

^bh^FRS: Fundamentals of Robotic Surgery.

^bi^ANFIS: adaptive neuro-fuzzy inference system.

^bj^DFT: discrete Fourier transform.

^bk^DCT: discrete cosine transform.

^bl^TSN: temporal segment network.

^bm^SORT: simple online and realtime tracking.

^bn^FCN: fully convolutional network.

^bo^UVA: urethro-vesicle anastomosis.

^bp^HMM: hidden Markov model.

^bq^ICO-OSCAR: International Council of Ophthalmology’s Ophthalmology Surgical Competency Assessment Rubric.

^br^EMG: electromyography.

^bs^COF: clearness of operating field.

**Table 4:** Summary of included studies on gesture and phase recognition.

Study	Year	Data modality	Data source	Sample size	ML^a^ method	Data availability
[78]	2025	Video	Microsurgery videos	132 sutures (11 surgeons)	T-LRCN^b^; ConvLSTM^c^	Private
[79]	2025	Video	Clinical (robotic prostatectomy)	N/A^d^	CNN^e^	Private
[80]	2025	Video	Clinical (cholecystectomy)	N/A	Action recognition model	Private
[81]	2024	Video	Clinical (laparoscopic pancreatoduodenectomy)	69 videos	CNN (TSM^f^-based)	Private
[82]	2023	Video	Simulator (low-cost appendectomy trainer)	43 videos (12 experts, 31 residents)	CNN (ResNet50^g^); Bi-LSTM^h^	Private
[83]	2023	Video; hand pose	Open surgery simulator	100 videos (25 clinicians)	MS^i^-TCN++^j^; I3D^k^; YOLOX^l^	Public
[13]	2022	Video	JIGSAWS^m^	39 videos (8 surgeons)	Calibrated MS-TCN; I3D; SlowFast	Public
[84]	2022	Video; kinematics	JIGSAWS; RARP-45	39 (JIGSAWS); 45 (RARP)	MA^n^-TCN	Public, private
[85]	2022	Video	Clinical (laparoscopic TAPP hernia repair)	119 videos (11 surgeons)	TeCNO^o^ (MS-TCN); HMM^p^; SV-RCNet^q^; KNN^r^	Private
[86]	2021	Video; kinematics	JIGSAWS; dVRK^s^ simulator (peg transfer)	75 videos (JIGSAWS); 24 sequences (dVRK)	Relational GCN^t^ (MRG-Net^u^); ResNet-18; TCN; LSTM	Public, private
[5]	2021	Video; optical flow	Clinical (robotic prostatectomy)	511 clips	AlexNet; LSTM; ConvLSTM	Private
[28]	2021	EEG^v^	Clinical (robotic prostatectomy)	34 surgeries (5 surgeons)	Extra Trees; RF^w^; bagging; KNN	Private
[87]	2020	Kinematics	JIGSAWS	39 trials (8 surgeons)	Multitask Bi-LSTM	Public
[14]	2020	Video	Clinical (laparoscopic colorectal)	300 videos	CNN (Xception); U-Net^x^	Private
[88]	2019	Kinematics	JIGSAWS; V-RASTED^y^	8 (JIGSAWS); 18 (V-RASTED)	Time-delay neural network (TDNN^z^)	Public

^a^ML: machine learning.

^b^T-LRCN: temporal long-term recurrent convolutional network.

^c^ConvLSTM: convolutional long short-term memory.

^d^N/A: not available.

^e^CNN: convolutional neural network.

^f^TSM: temporal shift module.

^g^ResNet50: Residual Network With 50 Layers.

^h^Bi-LSTM: bidirectional long short-term memory.

^i^MS: multistage.

^j^TCN: temporal convolutional network.

^k^I3D: inflated 3D convolutional network.

^l^YOLOX: You Only Look Once X.

^m^JIGSAWS: Johns Hopkins University and Intuitive Surgical Inc Gesture and Skill Assessment Working Set.

^n^MA-TCN: multiaxis temporal convolutional network.

^o^TeCNO: temporal convolutional network for operating room phase recognition.

^p^HMM: hidden Markov model.

^q^SV-RCNet: surgical video recurrent convolutional network.

^r^KNN: k-nearest neighbors.

^s^dVRK: da Vinci Research Kit.

^t^GCN: graph convolutional network.

^u^MRG-Net: multirelational graph network.

^v^EEG: electroencephalography.

^w^RF: random forest.

^x^U-Net: U-shaped convolutional network.

^y^V-RASTED: Virtual Reality Assessment of Surgical Tool-Handling and Ergonomic Dexterity.

^z^TDNN: time-delay neural network.

**Table 5:** Summary of included studies on real-time assistance and safety analysis.

Study		Year	Data modality	Data source	Sample size	ML^a^ method	Data availability
**Real-time feedback/guidance**							
	[89]	2025	Video	Clinical (robotic mastectomy)	10 surgeries	Dissection plane segmentation model	Private
	[90]	2025	Video	Clinical (colorectal)	N/A^b^	Nerve recognition model	Private
	[91]	2024	Video	Clinical (pituitary surgery)	>200 videos	Mask R-CNN^c^	Private
	[3]	2024	Video	Clinical (robotic urology)	2 surgeries	Video transformer network	Private
	[92]	2023	Video (multicamera)	Laparoscopic box-trainer (Fundamentals of Laparoscopic Surgery peg transfer)	55 videos; 5000+ images (9 participants)	SSD^d^ (ResNet50^e^ V1 FPN^f^); rule-based sequential assessment	Public
	[57]	2022	Video (top-down cameras)	Laparoscopic box-trainer	55 videos (9 residents)	SSD; ResNet50; rule-based	Public
	[29]	2022	Video; kinematics	dVRK^g^ simulator (SurRoL)	>1000 trials	Reinforcement learning (RL^h^)	Private
	[93]	2022	Video	Clinical (cholecystectomy)	290 surgeries	Semantic segmentation (ResNet50+PSPNet^i^)	Private
**Tool tracking/pose estimation**							
	[94]	2025	Video	Laparoscopic simulator	10 surgeons	DeepLabCut (Pose Estimation)	Private
	[15]	2025	Video	3D printed model	15 procedures	YOLOv8^j^; BoT^k^-SORT^l^	Private
	[95]	2024	Video; IMU	SutureCoach Simulator	81 participants	CNN^m^ (ResNet-50)	Private
	[23]	2023	Video; kinematics	Custom Multiprocedure Dataset	950 frames (5 surgeries)	GCN^n^; ResNet; Transformer	Private
	[96]	2022	Video (simulated)	da Vinci simulator	9 videos	6-DoF^o^ Motion Planning	Private
	[97]	2022	Video (synthetic)	Synthetic MICCAI^p^; JIGSAWS^q^	8 videos	PnP^r^; optical flow; time series forest	Public
	[98]	2022	Video	ATLAS Dione^s^; Endovis^t^; Cholec80^u^	99 clips + 1083 frames	DSCNet-CLSTM^v^; CNN regression	Public
	[31]	2021	Video	Clinical (cataract surgery); CaDIS^w^; Cataract-101	629 images	Faster R-CNN; SSD; RetinaNet; Scaled-YOLOv4	Public, Private
**Error detection/safety**							
	[99]	2024	Video	JIGSAWS	N/A	Transformer	Public
	[100]	2024	Video	Microsurgery simulator	24 trials (12 surgeons)	Semantic segmentation (ResNet-50)	Private

^a^ML: machine learning.

^b^N/A: not available.

^c^R-CNN: region-based convolutional neural network.

^d^SSD: single shot detector.

^e^ResNet: residual network.

^f^FPN: feature pyramid network.

^g^dVRK: da Vinci research kit.

^h^RL: reinforcement learning.

^i^PSPNet: pyramid scene parsing network.

^j^YOLO: You Only Look Once.

^k^BoT: bag of tricks.

^l^SORT: simple online and realtime tracking.

^m^CNN: convolutional neural network.

^n^GCN: graph convolutional network.

^o^6-DoF: 6 degrees of freedom.

^p^MICCAI: Medical Image Computing and Computer Assisted Intervention.

^q^JIGSAWS: Johns Hopkins University and Intuitive Surgical Inc Gesture and Skill Assessment Working Set.

^r^PnP: perspective-n-point.

^s^ATLAS Dione: Applied Technology Laboratory for Advanced Surgery Dione dataset.

^t^EndoVis: Endoscopic Vision Challenge.

^u^Cholec80: a dataset of 80 cholecystectomy procedure videos.

^v^DSCNet-CLSTM: depth-wise separable convolutional network with convolutional long short-term memory.

^w^CaDIS: cataract dataset for image segmentation.

**Table 6:** Summary of included studies on surgical workflow and data analysis.

Study			Year		Data modality		Data source		Sample size		ML^a^ method	Data availability
**Learning curve analysis**												
	[101]	2024		Video		Simulator		N/A^b^		3D CNN^c^; TSN^d^		Private
	[102]	2022		Kinematics; force		VR^e^ simulator (NeuroVR)		50 participants		KNN^f^ (for metric selection); ANOVA		Private
**Video retrieval/content understanding**												
	[26]	2024		Image; text (VQA^g^)		EndoVis17/18; DAISI^h^-VQA		545 QA^i^ pairs (DAISI)		Multimodal LLM^j^ (InstructBLIP); CL^k^		Public
	[103]	2024		Video		Cholec80^l^; Cataract-101; CholecT45^m^		80, 101, 45 videos		Hashing; Transformer		Public
	[25]	2022		Video		Surgical Actions 160; Cataract-101		160 clips; 101 surgeries		CNN; LSTM^n^; TCN^o^; contrastive learning		Public
	[104]	2019		Kinematics		JIGSAWS^p^		8 participants		DTW^q^; DBA^r^; NLTS^s^		Public

^a^ML: machine learning.

^b^N/A: not available.

^c^CNN: convolutional neural network.

^d^TSN: temporal segment network.

^e^VR: virtual reality.

^f^KNN: k-nearest neighbors.

^g^VQA: visual question answering.

^h^DAISI: Database for AI Surgical Instruction.

^i^QA: question-answer.

^j^LLM: large language model.

^k^CL: contrastive learning.

^l^Cholec80: a dataset of 80 cholecystectomy procedure videos.

^m^CholecT45: a dataset of 45 cholecystectomy procedure videos with triplet annotations.

^n^LSTM: long short-term memory.

^o^TCN: temporal convolutional network.

^p^JIGSAWS: Johns Hopkins University and Intuitive Surgical Inc Gesture and Skill Assessment Working Set.

^q^DTW: dynamic time warping.

^r^DBA: DTW barycenter averaging.

^s^NLTS: nonlinear time series.

### AI-Based Methods for Surgical Skill Assessment

#### Overview

Across the 92 papers, there’s a wide range of ML techniques used to classify surgical movements, recognize gestures, or assess technical skill (Table 7). The dominant approach involved CNNs, appearing in 55 (59.8%) studies. This broad category includes 2D networks such as ResNet for feature extraction, spatiotemporal models such as Inflated 3D ConvNet (I3D) for video analysis, and temporal convolutional networks (TCNs) for sequence modeling [72,73]. Classical ML methods also remained prevalent, with 29 (31.5%) studies using techniques such as support vector machine (SVM), random forests, and regression models, particularly for skill classification from handcrafted features [24,56]. Object detection and segmentation models (eg, You Only Look Once[YOLO] and U-shaped convolutional network [U-Net]) were specifically used in 16 (17.4%) studies to track instruments or identify anatomy [14,57]. Recurrent neural networks (RNNs), primarily LSTMs, were featured in 14 (15.2%) studies to capture temporal dependencies [5]. More recent architectures based on transformers and attention were used in 11 (12%) studies [3,9], while other approaches included time series and probabilistic methods (eg, dynamic time warping or hidden Markov models [HMM]) in 9 (9.8%) studies [104] and unsupervised or self-supervised learning in 7 (7.6%) studies [32,66].

**Table 7:** Frequency of different machine learning (ML) techniques across the 92 reviewed papers. Convolutional neural network (CNN)–based architectures were the most common. The total count exceeds 92 because many studies used multiple techniques (eg, a CNN for feature extraction and a long short-term memory (LSTM) for temporal analysis).

Frequency of machine learning techniques used	Studies (n=92), n
Unsupervised & self-supervised learning	7
Time series & probabilistic methods (DTW^a^, HMM^b^, etc)	9
Transformers & attention mechanisms	11
RNN^c^-based architectures (LSTM, etc)	14
Object detection and segmentation (YOLO^d^, U-Net^e^, etc)	16
Classical ML (SVM^f^, random forest, KNN^g^, etc)	29
CNN-based architecture (ResNet^h^, I3D^i^, TCN^j^, etc)	55

^a^DTW: dynamic time warping.

^b^HMM: hidden Markov model.

^c^RNN: recurrent neural network.

^d^YOLO: You Only Look Once.

^e^U-Net: U-shaped convolutional network.

^f^SVM: support vector machine.

^g^KNN: k-nearest neighbors.

^h^ResNet: residual network.

^i^I3D: inflated 3D convolutional network.

^j^TCN: temporal convolutional network.

#### Classical ML

Across the reviewed literature, 29 (31.5%) studies used classical ML methods such as SVMs, random forests, and k-nearest neighbors. These models were typically applied to features extracted from kinematics data or motion trajectories such as path length, speed, and jerk. For instance, Ebina et al [56] applied support vector regression to predict Global Objective Assessment of Laparoscopic Skills (GOALS) scores from motion capture data, while Hasani et al [63] used a k-nearest neighbors classifier on kinematic features to assess suturing skill.

Separately, 9 (9.8%) studies used time-series, probabilistic, or rule-based models to address temporal dependencies and uncertainty. Gorantla and Esfahani [74], for example, used motor control descriptors as input to HMMs, demonstrating that structured, time-aware models could infer meaningful surgical patterns even with limited data. To tackle label ambiguity directly, Iranfar et al [52] incorporated Dempster-Shafer theory to quantify classification uncertainty. Their method fused soft evidence and handled ambiguous gesture transitions more robustly than standard decision boundaries, highlighting the value of belief-based reasoning when surgical labels are imprecise or noisy.

While these methods provided valuable early insights, their reliance on handcrafted features and limited scalability led to a broader adoption of deep learning models capable of learning directly from raw, high-dimensional inputs.

#### Deep Learning Models

Deep learning has become the dominant approach in recent years, with various architectures used to learn from high-dimensional data. The most common technique, involving CNNs, appeared in 55 (59.8%) studies. This was often complemented by RNNs in 14 (15.2%) studies, and more recently, transformers and attention-based models in 11 (12%) studies.

CNNs were commonly applied to video data or spatially structured kinematic sequences. A primary application was in object detection and segmentation, which was central to 16 (17.4%) studies. For instance, Nakajima et al [36] used a CNN-based semantic segmentation model to estimate the dissected tissue area from laparoscopic colorectal videos, allowing a phase-specific efficiency metric that correlated with surgical expertise. Beyond segmentation, CNNs were used for end-to-end skill assessment. For example, Jian et al [69] implemented a multitask CNN framework for video-based surgical skill assessment, jointly learning to predict gesture classes and global skill labels. Similarly, Tanin et al [30] used an ensemble of 2D and 3D CNNs to assess skill across cataract surgery phases using only endoscopic video, demonstrating the feasibility of sensor-free performance evaluation. Some work focused on temporal convolutions, such as Menegozzo et al [88], who applied a time-delay neural network to robotic kinematic data for real-time gesture segmentation during knot tying, showcasing the early use of temporal convolutional models for skill-related sequence modeling. Yanik et al [101] further advanced this by developing a deep learning pipeline to track surgical skill acquisition over time using only laparoscopic training videos. Their model combined 3D CNNs with temporal segment networks to classify skill levels across repeated training sessions, capturing performance trajectories without relying on external sensors. The approach demonstrated strong accuracy in distinguishing novice from experienced participants and offered a scalable solution for monitoring skill progression in simulation-based curricula.

For sequential modeling, 14 (15.2%) studies used RNN variants, such as LSTM networks and gated recurrent units. These models processed frame-level or kinematic data over time to detect transitions between surgical gestures or estimate proficiency [5,32]. However, they often required fixed-length inputs and struggled with long-range dependencies.

To address these limitations, hybrid architectures such as long-term recurrent convolutional networks have gained traction by combining spatial and temporal representations more effectively. Recent implementations leverage variations such as convolutional long short-term memory (ConvLSTM), temporal pooling, and 2-stream fusion to improve fine-grained subphase classification and enable skill assessment. These models not only improve accuracy but also provide task-aware metrics such as phase-wise confidence or timing, offering interpretable insights into surgical performance. While such architectures perform well even in challenging video-only setups, they often remain constrained by unimodal inputs and require careful handling of visually similar transitions [78].

Reflecting a recent trend in sequence modeling, 11 (12%) studies introduced transformer-based models to leverage their strength in capturing long-range dependencies. For example, Zuluaga et al [3] applied a video transformer network for real-time procedural annotation during robotic-assisted prostatectomy, demonstrating high alignment with expert labels and strong temporal generalization. Zhang et al [9] used a continuous wavelet transform-vision transformer (ViT) architecture combining continuous wavelet transforms and vision transformers to assess suturing skill, achieving over 90% classification accuracy on the JIGSAWS dataset. Compared to RNNs, transformers offered better parallelization and interpretability through attention weights, making them attractive for real-time surgical guidance applications.

#### Self-Supervised and Domain Adaptation Methods

As labeling surgical data is time-consuming and often subjective, 7 (7.6%) studies explored self-supervised, semisupervised, or transfer learning strategies to reduce dependence on annotated datasets. These methods include contrastive learning to differentiate between samples [50], reinforcement learning for generating optimal trajectories [29], and pseudolabeling to leverage unlabeled data [16].

Several studies focused on domain adaptation to improve generalizability across different surgical tasks. For example, Wang et al [16] developed a domain-adaptive model that used uncertainty-aware self-supervised learning to generate pseudolabels for an unlabeled surgical task. This allowed the model to generalize from the JIGSAWS dataset to a novel VR simulation without requiring new annotations. Similarly, Alkadri et al [10] improved skill classification accuracy by applying transfer learning from a model trained on a similar surgical task, demonstrating its effectiveness on small datasets.

In a different approach, Anastasiou et al [50] applied contrastive regression within a transformer architecture, where a surgical video was scored based on its deviation from an expert reference performance, providing a fine-grained and interpretable assessment.

#### Multimodal and Graph-Based Approaches

To capture the complexity of surgical actions, 12 (13%) studies integrated multiple data streams—such as video, kinematics, and biosignals—within unified deep learning architectures [45,59,84]. These multimodal systems aim to improve robustness by fusing complementary performance perspectives, with techniques ranging from simple feature concatenation to more sophisticated attention and graph-based methods. A common strategy involves using attention mechanisms to dynamically weigh information; for instance, Amsterdam et al [84] developed a multimodal attention TCN that dynamically fuses video and kinematic features, allowing the model to rely more on one modality when the other is compromised (eg, by visual occlusion).

A prominent direction in this space is the use of graph-based learning for structural alignment across modalities [23,86]. Long et al [86] designed a relational graph network that jointly processes visual and kinematic embeddings to enhance gesture recognition. Their model treats video, left-hand kinematics, and right-hand kinematics as distinct nodes in a graph, refining their representations through relation-aware message passing that models concepts such as “vision-to-motion” and “in-between-motions” [86]. In a different application, Liu et al [23] introduced a visual-kinematics graph learning framework focused specifically on instrument tip segmentation. Their model constructs directed graphs from both image features and rendered kinematic silhouettes, using graph convolutional networks (GCNs) and a node-wise contrastive loss to enable procedure-agnostic segmentation [23].

These architectural strategies reflect a broader shift toward models that internalize surgical scene structure. Graph-based representations capture spatial relationships between instrument components or modalities [23,86], while attention mechanisms dynamically filter relevant signals across time [84]. Compared to early fusion techniques that rigidly concatenate inputs, these flexible approaches are better suited to cope with occlusions, variable motion dynamics, and visual noise in clinical environments [84,86].

### Data Modalities and Multimodal Fusion

#### Overview

An analysis of the 92 included studies reveals 3 primary data modalities used for surgical skill assessment. The most common approach, found in 58 (63%) studies, was the use of video-based methods that analyze endoscopic or external camera footage. This was followed by 18 (19.6%) studies that relied on kinematic-only data from robotic systems or sensors. Finally, 12 (13%) studies used multimodal approaches, fusing 2 or more data streams to create more robust models.

#### Kinematic-Only Studies

Kinematic data—comprising tool tip positions, velocities, forces, and trajectory-derived metrics—was a foundational modality, particularly in studies using robotic systems or standardized datasets such as JIGSAWS [32,72]. Early studies in this area focused on handcrafted features such as motion smoothness and jerk [63]. More recent work has applied deep learning directly to kinematic sequences; for example, Anh et al [32] compared 9 different feature extraction techniques, including CNN and LSTM, for evaluating skills in near real time from robotic motion data.

Building on the pursuit of interpretable motion features, Shayan et al [37] applied functional data analysis to hand rotation signals captured during open suturing. Using functional principal component analysis and functional ANOVA, they extracted temporally structured features that significantly distinguished expert from novice performance. This approach highlights the potential of statistical modeling for capturing subtle skill differences while preserving interpretability.

Soleymani et al [48] introduced an information-theoretical framework to quantify bimanual coordination through mutual information and dynamic time warping between hand trajectories. By analyzing peg transfer tasks, they demonstrated that expert surgeons exhibited higher levels of interhand coordination, reinforcing the value of motion coupling as a skill indicator. Their method adds a relational perspective to kinematic analysis and aligns with the broader trend toward interpretable and quantifiable skill metrics.

#### Video-Based Methods

Video-based methods use endoscopic or external surgical footage to extract visual features for gesture recognition, procedural phase segmentation, or skill evaluation. These approaches typically rely on CNNs [5,54] to extract spatial patterns from frames, or on temporal models such as 3D-CNNs and transformers [3,73] to handle motion and sequence information. In particular, transformer-based models have gained traction due to their ability to capture long-range dependencies and provide interpretable attention maps, which are useful for real-time applications and explainability. Recent work by Guo et al [41] introduced a multitask transformer (multitask vision transformer [MT-ViT]) capable of both phase recognition and skill assessment from streaming long-form surgical videos. Their model handled real-time inference over continuous video using temporally local attention blocks, achieving competitive accuracy across both classification and regression tasks.

One important axis of variation is the application setting. In simulation-based environments, models often benefit from standardized camera views and task repetition. Liu et al [23] integrated video features with motion data using a spatiotemporal attention model, enhancing gesture recognition in a bench-top robotic setup. Their study also noted that performance gains were partially attributed to clean and well-labeled synthetic datasets, raising concerns about generalizability to more variable clinical data. In a similar direction, Erlich-Feingold et al [34] conducted a pilot study using deep learning to classify surgeon skill levels from video recordings of laparoscopic simulations. Their model, trained on simulated tasks involving novice and expert participants, achieved high agreement with human raters, particularly in identifying novice-level performance. While limited to a simulation setting and video-only input, this work reinforces the role of AI in formative assessment and early-stage skill differentiation, highlighting its practical relevance for surgical training programs. Another approach to simulation-based feedback involves extracting interpretable kinematic proxies from video alone. For instance, recent work estimated hand roll angles in suturing tasks using CNN-based vision models trained on hand-segmented frames [95]. The predicted “number of rolls”—a well-established metric for suturing fluency—successfully distinguished expertise levels in simulator environments, offering a low-cost and contactless method of skill assessment without requiring wearable sensors. This direction highlights how proxy motion cues derived from video can support scalable assessment pipelines when direct kinematics are unavailable.

In contrast, clinical studies often work with noisier, lower-frame-rate data and fewer labeled samples. Saricilar et al [54] trained CNNs on laparoscopic gastrectomy recordings, showing that even small datasets can be useful when phase boundaries are well annotated. They emphasized that transfer learning from simulation-trained models significantly boosted performance in data-scarce clinical environments. Zuluaga et al [3] demonstrated real-time procedural annotation using transformer models on robotic-assisted urological procedures, bridging the gap between offline learning and intraoperative support. Recent work has also leveraged deep action recognition models to directly link surgical step classification with surgeon proficiency. Yen et al [80] trained a model to recognize key actions—such as dissecting, exposing, and cutting—in laparoscopic cholecystectomy, showing that the predicted action patterns correlated significantly with GOALS-based competency scores. Their annotated video dataset, created from real clinical procedures, adds to the growing pool of surgical video benchmarks designed for both action understanding and skill assessment.

Following this trend of direct video-to-score pipelines, Yeh et al [39] developed PhacoTrainer, an AI tool trained on annotated cataract surgeries that achieved high agreement with expert Objective Structured Assessment of Cataract Surgical Skill ratings. Its design focused on efficiency and skill differentiation in ophthalmic microsurgery, reinforcing the feasibility of end-to-end assessment in standardized clinical workflows. Tanin et al [30] proposed an ensemble of 2D and 3D convolutional networks to classify surgical skill levels in cataract surgery using raw video data. Their architecture combined time-distributed 2D CNNs with LSTM layers and a parallel 3D CNN stream, enabling skill prediction across multiple phases without requiring external sensors or tool annotations. This study highlights the feasibility of pure vision-based assessment in ophthalmic microsurgery, although its generalizability to other surgical contexts remains to be validated.

Similar efforts in microsurgical contexts have explored long-term recurrent convolutional network–based architectures for both phase recognition and skill assessment. By analyzing short video segments using hybrid recurrent-convolutional models, these methods can estimate both the likelihood of a subphase and the confidence or duration associated with its execution. This dual-metric evaluation enables more nuanced feedback—distinguishing expert control from novice hesitation—even in the absence of kinematic or tool-tracking data. Such techniques show particular promise for vision-only feedback systems in training environments, though their robustness under varying visual conditions remains an open question [78].

Beyond standard frame-level classification, several studies investigated specialized visual features to improve robustness in surgical settings. For example, sparse optical flow has been used to derive minimal yet informative motion descriptors from video sequences [64]. Others have proposed evaluating surgeon performance based on field-of-view clarity as a surrogate for procedural control and efficiency [77]. Pose estimation techniques—derived from articulated tool tracking or skeleton inference—were also applied to augment gesture classification in video-only settings [97], particularly where direct kinematic data were unavailable. Building on this, Fukuta et al [94] used DeepLabCut-based pose estimation in a structured simulator environment, tracking laparoscopic forceps trajectories using top-down video. Unlike Elek et al [97], who focused on skeleton-based gesture inference, Fukuta et al’s [94] approach emphasizes fine-grained tool motion tracking for skill validation and feedback. Their system demonstrates the promise of simulation-calibrated vision-only methods, especially when domain constraints (eg, lighting or tool occlusion) can be controlled to improve tracking stability.

Moreover, deep segmentation models have been explored to extract fine-grained surgical signals from video, such as vessel displacement during microvascular suturing [100]. Tang et al [100] also noted that segmentation-driven features enabled interpretable feedback, allowing for localized performance assessment rather than coarse global scores. These approaches suggest that video alone, when processed with task-specific priors, can provide insight into subtle aspects of surgical technique.

While video offers richer contextual cues—such as tool-tissue interaction and field visibility—it introduces challenges including variable lighting, occlusion, and camera motion. As a result, several studies combine video with additional modalities such as kinematics or pose estimation to stabilize predictions and reduce uncertainty, particularly in real-world surgical environments.

#### Multimodal Fusion

Multimodal approaches combine distinct data types—such as video, kinematics, force, and electroencephalography—to provide a more comprehensive representation of surgical performance. These methods aim to leverage the complementary nature of different modalities to improve classification accuracy, interpretability, and resilience in real-world scenarios.

Fusion strategies typically fall into 3 categories: early fusion (input-level concatenation), late fusion (decision-level), and intermediate fusion using attention or graph-based mechanisms. Luongo et al [5] demonstrated that integrating video and kinematics within an LSTM architecture yielded superior gesture segmentation compared to unimodal models. Similarly, Liu et al [23] implemented a 3D CNN with temporal attention fusion to combine spatiotemporal cues from video and motion signals, leading to more stable predictions in robotic tasks. However, they also reported latency-performance trade-offs, posing challenges for deployment in time-critical HAT applications. Time-synchronized multimodal systems have also been explored for procedural understanding. For example, Fawaz et al [104] used aligned video and kinematic streams to improve surgical phase segmentation accuracy on benchmark datasets.

Force sensing is another valuable modality in surgical simulation. Alkadri et al [4] used position and force metrics from a VR simulator to train a multilayer perceptron classifier, identifying key skill-related features through feature selection. Their work emphasized interpretable modeling and efficiency, though performance was demonstrated primarily on a single simulator task. In a follow-up study, the same group explored transfer learning with force and motion metrics in a hybrid VR/augmented reality (AR) simulator [10]. While transfer learning improved test accuracy, the study acknowledged the need to address modality shifts and environmental variability for broader generalization. Takács and Haidegger [68] developed an adaptive neuro-fuzzy inference system using force-based metrics across multiple training tasks, producing task-specific subscores for skill assessment. Multiview and multi-instrument tracking systems have been developed to capture fine-grained tool behavior across training and clinical environments, expanding the coverage and fidelity of motion-based assessment [70].

Some studies explored cognitive and biometric signals to expand beyond physical motion. Natheir et al [2] used electroencephalography signals during neurosurgical simulation to train a semisupervised artificial neural network (ANN), achieving high accuracy in differentiating skill levels. However, their approach exhibited limited generalizability outside neurosurgical contexts, indicating a need for task-agnostic models. Shafiei et al [45] combined electroencephalography and eye-tracking data from live animal surgeries and applied ensemble models to classify subtasks and skill levels based on both physiological and behavioral metrics. Their study emphasized cognitive load inference as a critical component for adaptive HAT feedback. In a related effort, Shafiei et al [28] demonstrated that electroencephalography signals alone could be used to classify surgical gestures, using features derived from functional brain networks and power spectral densities. This work provided early evidence linking cognitive state and motor intention in surgical performance modeling. Gesture tracking using temporal alignment techniques—such as dynamic time warping—has also been proposed to accommodate variations in execution pace and movement timing [62].

Bkheet et al [83] used pose estimation combined with I3D features to inform gesture segmentation models in open surgery training videos, enabling interpretable skill proxy-based feedback on hand-tool interaction. Singh et al [47] captured multisensor motion data using electromagnetic tracking and inertial measurement units to extract fine motion metrics such as angular change and path length during open suturing. Despite providing high-fidelity motion data, Singh et al [47] reported calibration drift and alignment issues, posing challenges for long-duration tasks.

### Benchmarks and Validation Practices

#### Overview

Benchmarking plays a central role in evaluating and comparing ML models for surgical skill assessment. However, the landscape is fragmented, with diverse datasets, inconsistent evaluation criteria, and limited external validation. This section examines the most commonly used datasets, assessment standards, evaluation metrics, and the methodological challenges that influence reproducibility and generalizability in the literature.

#### Datasets Used

The JIGSAWS dataset was the most frequently used benchmark, providing synchronized video and kinematic data for 3 basic tasks (suturing, knot tying, and needle passing) [9,12,63,66,67,69,72,84]. However, JIGSAWS is limited by its low task diversity and controlled recording environment, making it less representative of real-world surgical variability. Despite these limitations, it enabled classification and regression models to evaluate motion-based surgical skill in controlled environments. Other datasets included Cholec80 [58,80,103], Cataract-101 [25,60], EndoVis [26,98], LapSig300 [14], and various simulated VR platforms [4,10,47,59,68]. These datasets vary widely in data types, incorporating video-only streams, kinematics, tool usage, or even cognitive signals such as electroencephalography and eye tracking [27,45]. The EndoVis challenge series provided a standardized benchmark for segmentation and pose estimation tasks but lacks longitudinal datasets necessary for tracking surgeon progression over time. LapSig300, in contrast, offers specialized tool vibration signals for laparoscopic task analysis, representing a more niche application. Simulator-based systems incorporating AI-driven validation and feedback loops have also been explored, contributing to benchmark creation and structured training platforms [94]. However, studies such as Alkadri et al [4,10] highlighted the domain gap challenges when transferring models trained on simulator data to clinical environments, underscoring the need for robust domain adaptation techniques. Additionally, electroencephalography and eye-tracking datasets remain limited by small sample sizes and are often restricted to animal or phantom studies, raising concerns about generalizability to human surgical performance. [27,45]

#### Skill Assessment Standards

Multiple studies used structured scoring systems such as OSATS, GRS, or Endoscopic Surgical Skill Qualification System (ESSQS) [42,43,51,66,69,72]. However, studies such as Komatsu et al [43] reported significant interrater variability in ESSQS scoring, raising concerns about reproducibility in subjective assessments. Some adopted task-specific rubrics such as International Council of Ophthalmology’s Ophthalmology Surgical Competency Assessment Rubric for ophthalmic microsurgery [75], or GOALS for laparoscopic tasks [49]. Labels were often derived from expert review, standardized rubrics, or thresholds applied to continuous scores (eg, binarizing OSATS/GRS for classification [71]). Hoffmann et al [42] introduced a hybrid approach by combining rubric-based scores with automated kinematic metrics, aiming to balance interpretability with objectivity. Several works used self-reported skill level [53] or pass/fail outcomes [27]. Pan et al [53] observed discrepancies between self-reported skill levels and objective performance metrics, highlighting limitations in self-assessment reliability. Others generated derived metrics from kinematic analysis [47,61,63]. Classification frameworks based on explicit gesture features have also been evaluated, providing a more interpretable bridge between visual motion and standardized rubrics [40]. Chen et al [40] further enhanced interpretability by incorporating attention-based visualization maps, supporting explainable AI in surgical skill assessment.

#### Evaluation Metrics

Model performance was evaluated using accuracy, *F*_1_-score, and area under the curve (AUC) for classification tasks [43,58,66,73,75]. Komatsu et al [43] further used confusion matrices to diagnose class-wise prediction errors, aiding in understanding model misclassifications. Fathollahi et al [58] highlighted that AUC and *F*_1_-scores provided more reliable performance indicators in the presence of imbalanced class distributions, a common challenge in surgical datasets. Regression-based systems predicting continuous skill scores used metrics such as mean absolute error (MAE), root-mean-square error, and Spearman correlation [10,23,72]. Liu et al [23] emphasized that Spearman correlation better captured subjective skill ranking alignments compared to absolute error metrics such as MAE, making it particularly suitable for rubric-based assessment. Interrater agreement and expert consensus were also reported for label consistency [65,85], with Cohen κ or intraclass correlation coefficient values supporting annotation reliability [81]. Lavanchy et al [65] observed reduced interrater agreement in complex procedural annotations, underscoring the influence of task complexity on labeling consistency. You et al [81] advocated for standardized annotation protocols to enhance interrater reliability, particularly when aggregating multi-institutional datasets. In addition, motion analysis techniques applied to wet-laboratory laparoscopic training environments have shown measurable skill discrimination, supporting their inclusion as quantitative ground truths [56]. Recently, Singh et al [46] introduced the Smoothness of Force metric, derived from time- and frequency-domain analysis of force signals during simulated tissue handling. Their results showed strong skill-level separation, suggesting that signal-based smoothness measures may serve as low-cost, interpretable indicators for assessing manipulation quality. However, Ebina et al [56] cautioned that such metrics are context-sensitive, limiting their generalizability beyond specific task types.

#### Challenges in Benchmarking

Despite growing datasets, benchmarking in surgical AI remains inconsistent. The size and diversity of available datasets are still limited, particularly in real-world procedures. Alkadri et al [10] noted that leave-one-user-out (LOUO) validation becomes challenging when user-specific data is scarce, exacerbating class imbalance issues. Labels may be subjective or derived from inconsistent evaluation standards, leading to variability in reported outcomes. Validation strategies vary, with LOUO and cross-validation applied unevenly across studies [10,16,24,73]. Wang et al [16] emphasized that inconsistent reporting of model inputs and preprocessing pipelines further hinders reproducibility across studies. Only a few works have tested models across institutions, tasks, or input modalities [3,66,85], which limits generalization. Zuluaga et al [3] identified mismatches in input modality characteristics as a major barrier to generalizing models trained on homogeneous datasets. Takeuchi et al [85] further highlighted interinstitutional annotation drift, which complicates benchmarking consistency. Early studies also underscored the role of sensor-rich laparoscopic systems in capturing workflow states and expertise levels, laying the groundwork for modern validation strategies [76]. Improved benchmarking will require harmonized rubrics, open multicenter datasets, and transparent reporting of model inputs and validation logic. Alternative benchmarking tools, such as fuzzy-logic scoring engines for simulation-based peg transfer tasks, have been proposed to model uncertainty and provide flexible scoring interpretations [57,92]. Beyond uncertainty modeling, Fathabadi et al [92] demonstrated that fuzzy scoring systems enhanced interpretability, particularly in assessing novice-level performance with more nuanced feedback.

### From Assessment to Decision-Making: Toward HAT

#### Overview

While traditional research in surgical AI has focused on post hoc skill assessment, a growing number of studies are moving toward systems that support real-time interaction, adapt to users, and contribute to intelligent surgical guidance. This transition forms the foundation for HAT, in which AI systems not only evaluate but also actively collaborate with surgeons.

#### Real-Time and Feedback-Capable Models

Several studies developed models optimized for real-time inference and feedback. Anh et al [32] evaluated lightweight deep learning pipelines that prioritize low-latency inference, making them suitable for looped skill-feedback systems, though not without trade-offs in accuracy. Recent transformer-based methods have expanded this space by enabling on-the-fly labeling and procedural awareness. For instance, Zuluaga et al [3] deployed a transformer for robotic-assisted prostatectomies that delivered real-time procedural annotations, but reported difficulties in generalizing to surgical variations not seen during training. In a similar direction, Guo et al [41] introduced a multitask transformer that performs both phase segmentation and skill prediction over continuous video streams, supporting feedback that evolves with procedural progression. While these systems focus on task structure and performance cues, others have emphasized anatomy-aware guidance. Ryu et al [90] trained a deep learning model to identify nerves in laparoscopic colorectal video, enabling real-time overlays that support both intraoperative navigation and surgical education—especially critical in high-risk regions where visual precision informs safety. Similarly, Madani et al [93] used semantic segmentation to distinguish anatomical zones and hazard boundaries in cholecystectomy, offering interpretable heatmaps aligned with expert visual reasoning.

Khan et al [91] extended anatomy-aware AI to the neurosurgical domain by developing a mask region-based convolutional neural network–based model for recognizing critical structures during endoscopic pituitary surgery. Trained on a large dataset of over 2 million annotated video frames from 200 clinical procedures, their model achieved a mean average precision exceeding 0.8 across multiple anatomical labels, including the optic nerve and carotid artery. The study sets a high benchmark for anatomical segmentation quality and reinforces the role of AI in intraoperative safety and education. Although not yet deployed in real time, the scale, annotation rigor, and surgical relevance position it as a promising system for operative decision support.

Building on these anatomy-aware segmentation systems, Lee et al [89] developed a model to identify dissection planes during robotic mastectomy. Using annotated video frames extracted at 1-second intervals from 10 procedures, their deep learning pipeline produced accurate and interpretable visual overlays, achieving a mean Dice similarity coefficient of 0.78. Although limited by dataset size and the lack of real-time deployment, the system emphasizes clinically meaningful labels and frame-level precision, presenting a viable direction for embedding AI-based visual guidance into surgical education platforms. This aligns with the broader shift toward using intraoperative video not only for assessment, but also for training-aware assistive decision support. These approaches illustrate how AI can contribute to decision support without requiring gesture classification. Outside of vision-based overlays, motion-tracking systems have also advanced, such as Nwosu et al [15], who built a drill-tracking pipeline for otologic surgery that outputs motion smoothness and trajectory metrics with minimal latency, demonstrating practical utility in feedback-driven training scenarios.

These systems focus not only on accuracy, but also on speed and interpretability, critical components for intraoperative utility. Some models provided low-latency outputs by reducing input complexity or model depth [32]. Agarwal et al [13] used fine-tuned spatiotemporal backbones and a calibrated multistage TCN model to perform frame-level gesture segmentation on untrimmed surgical videos, achieving high segmental accuracy and edit distance scores. While not real-time, their approach demonstrates the importance of temporally precise modeling for gesture-aware feedback systems. Others aimed to balance latency and robustness by limiting model complexity or reducing input dimensionality [63]. Hasani et al [63] demonstrated that input dimensionality reduction was effective in enhancing model robustness, especially in noisy surgical environments.

#### Adaptive Systems and Sequential Decision-Making

Beyond inference speed, a second group of studies emphasized adaptability to different users, tasks, or conditions. These models incorporated temporal memory or personal context to adjust feedback and scoring dynamically.

Alkadri et al [10] trained ANN models on force and motion data in VR/AR simulators and applied transfer learning across procedures, indicating cross-task adaptability. However, they also acknowledged limitations due to domain shifts between simulated and clinical environments, which constrained full adaptation. Natheir et al [2] used semisupervised learning on electroencephalography and performance metrics to personalize skill estimation without full supervision. Their approach demonstrated the effectiveness of low-labeled data strategies in adapting models to individual users. Shafiei et al [45] integrated electroencephalography and eye-tracking in live robotic setups to detect workload and subtask transitions in real time. This integration improved system responsiveness to user cognitive states, enhancing its suitability for HAT scenarios.

Sequential decision-making models—such as TCNs or attention-based systems—were explored for gesture segmentation and phase tracking [9,23]. Liu et al [23] showed that temporal attention mechanisms significantly improved sensitivity to gesture transitions, improving the accuracy of real-time feedback. In a similar multitask framework, Guo et al [41] designed MT-ViT to output both phase segmentation and continuous skill scoring from video streams. The model’s sequential attention over long video segments allows it to track procedural progression while simultaneously inferring performance, supporting time-aware feedback across tasks. Sato et al [79] proposed a rule-based pipeline that extracts temporal dissection and exposure metrics by parsing instrument activation from the da Vinci surgical interface. Although their method does not involve learning-based adaptation, the extracted phase durations showed strong correlations with surgeon expertise, reinforcing the role of coarse temporal structure in modeling surgical performance. RNN-based architectures have also been applied in multitask settings, where systems simultaneously predict gesture class and procedural progress [87]. Amsterdam et al [87] further highlighted that such sequential outputs from these models enhance interpretability, offering users clearer insights into procedural state and system reasoning. These architectures allow systems to evolve predictions over time, a necessary feature for interactive and supportive autonomy.

Sequential reasoning has gained renewed interest with the application of transformer-based architectures tailored for surgical workflows. Building on the idea that decision-making in surgery often involves causally dependent gestures, recent models have introduced reasoning frameworks that emulate step-by-step thought processes. One such approach leverages gesture context to improve downstream error detection, highlighting the value of temporally grounded prompting within robotic video streams [99]. This design enables the system to anticipate and interpret surgical deviations by chaining likely action sequences—an advance that improves robustness and interpretability compared to traditional frame-based classifiers. Such methods suggest a move toward proactive AI systems that not only assess skill retrospectively but also reason through procedural intent in near real time.

#### Integration With Surgical Training and Guidance

Some works directly positioned their models within existing surgical education or robotic platforms. For example, Singh et al [47] used wearable sensors to provide motion quality metrics in open suturing, enabling dynamic coaching in physical training setups. Tonetti et al [49] designed a feedback system that maps model errors to OSATS subdomains, mirroring the structure of expert verbal guidance. Their rubric-aligned feedback approach notably enhanced trainee engagement by providing familiar, structured performance insights. Amsterdam et al [84] aligned transformer attention maps with human-defined gesture cues, facilitating interpretable handover of perceptual roles. This interpretability was shown to reduce cognitive load for trainees, making AI-assisted feedback more accessible. End-to-end video-based feedback systems have also been proposed for formative and summative evaluation within surgical curricula [55]. Yanik et al emphasized the scalability of such systems, supporting broader curricular integration.

In simulation environments, force-enabled feedback [4,68], visual annotations [3], or neural attention markers [23] were incorporated into intelligent tutoring systems or robotic task interfaces. Beyond technical feedback, some systems have prioritized scalability and accessibility. Bhatia et al [82] demonstrated the deployment of an AI-driven training system for low-resource environments, aiming to address instructor shortages in low- and middle-income countries and support foundational surgical education. Cruz et al [33] developed a scalable video-based AI platform that used YOLOv4 for laparoscopic instrument detection and feedback generation. Their model aligned performance scores with standardized surgical training frameworks and successfully replicated expert ratings across simulation tasks, showing the practical integration of real-time object detection in education pipelines. Liu et al reported that attention markers enhanced the granularity of localized feedback, improving the precision of corrective cues. Neurosurgical simulation has also benefited from AI-driven metric selection to track learning curves, helping to personalize instructional content and improve skill acquisition [102]. Ledwos et al [102] found that personalized metrics not only tailored instruction but also improved long-term skill retention. These developments mark early advances in shared control and multimodal feedback integration.

#### Gaps in Current HAT Readiness

Despite progress, few current systems achieve the robustness, adaptivity, and trust required for surgical HAT. Most studies rely on simulated tasks or preannotated data, limiting their ecological validity [12,66]. Generalization across users, procedures, and clinical setups remains underexplored, with only a handful testing multisite or cross-task robustness [10,16]. Wang et al [16] also reported inconsistencies in data pipelines and annotation protocols across clinical sites, further complicating generalization efforts. Recent clinical-phase models—for instance, in colorectal surgery—have shown that deep learning can accurately recognize procedural stages in real time. However, such tools remain scarce and largely confined to specialized domains [44]. Nakajima et al [44] emphasized that even when technically successful, these models face significant challenges in integrating with existing surgical workflows.

Interpretability is still treated as an afterthought in many deep models, although recent studies using SHAP [61] or attention visualization [84] have made promising strides. Yibulayimu et al [61] demonstrated improvements in local interpretability through SHAP summary and dependence plots and further implemented sample-by-sample force plots to provide real-time feedback during liposuction training. Similarly, Amsterdam et al’s [84] attention maps facilitated gesture-level insights but did not address higher-order procedural reasoning. True teaming will require models to communicate confidence, defer to the human when uncertain, and offer justifications in a format that fits surgical workflows.

In addition, almost no system integrates patient-specific variables or anticipates clinical decisions, key elements for future decision augmentation. Bridging the gap from skill scoring to surgical collaboration will require not only better models but better integration with the social, cognitive, and procedural context of the operating room.

## Discussion

### Principal Findings

This scoping review mapped recent advances in AI-based surgical skill assessment, focusing on methods that support HAT. Our synthesis addresses the 3 RQs posed in the Introduction:

#### RQ1: Adapting HAT Models for Real-Time Guidance and Personalized Feedback

The literature reveals a growing emphasis on real-time inference and feedback (one of our primary trends). This transition is critical for HAT adoption. Lightweight models and transformer-based architectures have enabled systems capable of intraoperative gesture recognition and phase annotation. Furthermore, emerging efforts toward adaptive and interpretable models align directly with the need for personalized guidance. Studies using SHAP explanations, attention visualizations, and personalized feedback pipelines represent initial steps toward systems that can communicate their reasoning and adjust to individual surgeon profiles. However, true real-time HAT remains limited: these systems are largely task-specific and constrained by generalization challenges across users, procedures, and clinical settings.

#### RQ2: ML Methods and the Enhancement of Multimodal Data

Our analysis confirms an evolution from unimodal to multimodal data integration, which serves to enhance classification and proficiency assessment. Multimodal approaches, combining kinematics, video, force, and biosignals, have been shown to enhance classification accuracy and robustness by leveraging complementary information sources. Techniques such as attention-based fusion and GCNs reflect this trend toward richer data representations, allowing models to process diverse data streams effectively. This validates the use of multimodal data as a key pathway to enhancing surgical movement classification for real-time applications.

#### RQ3: Benchmarks and Comparison With Expert Evaluations

The studies reviewed show that AI-driven skill assessment relies on a fragmented ecosystem of benchmarks. While standardized tools such as OSATS and GRS are widely used as ground truth for comparison, inconsistencies in evaluation criteria and validation practices remain a critical barrier. Validation strategies vary (eg, LOUO vs cross-validation), leading to variability in reported outcomes. Benchmarking is often constrained by simulation-based datasets (eg, JIGSAWS), which limits their ecological validity when comparing results to real-world expert evaluations. We conclude that standardized, harmonized protocols are essential to ensure the reproducibility and clinical relevance of AI assessments.

Together, these developments lay the foundation for transitioning from isolated assessment tools to intelligent surgical teammates. However, critical gaps remain before true HAT can be realized in surgical practice.

### Limitations and Research Gaps in Current Literature

Despite notable advancements, current AI-based surgical skill assessment methods face several limitations that hinder their readiness for HAT in real-world surgical environments. Our translational readiness analysis (Table 1) reveals that these limitations are not isolated shortcomings but stem from interconnected structural and methodological root causes.

First, ecological validity remains a significant concern. Most studies rely on simulation-based datasets or controlled laboratory environments, which do not fully capture the variability, complexity, and unpredictability of actual surgical procedures. Our TRL analysis confirms this pattern: 77.2% (71/92) of studies remain at TRL 3, indicating proof-of-concept validation on limited or benchmark data, while only 2.2% (2/92) have reached TRL 5 or above with clinical or cross-institutional testing. This concentration at low TRL levels reflects a fundamental bottleneck in the field: the scarcity of large-scale, annotated clinical datasets. Collecting and labeling intraoperative data requires expert time, institutional ethics approval, and standardized annotation protocols—resources that remain unevenly distributed across research groups [12,66]. This data scarcity also increases the risk of overfitting, particularly for deep learning architectures with high parameter counts trained on small, homogeneous datasets such as JIGSAWS (39 trials from 8 participants). High reported accuracies on such benchmarks may therefore reflect memorization of dataset-specific patterns rather than genuine learning of transferable surgical skill features.

Second, generalization across users, tasks, and clinical settings is underexplored. Our analysis shows that 38% (35/92) of studies lack any form of external validation, and only 15.2% (14/92) have conducted cross-site testing. The root causes are twofold: (1) pipeline inconsistencies and annotation discrepancies across institutions complicate robust evaluation [10,16,85], and (2) domain shift between simulated and clinical environments degrades model performance when training and deployment conditions differ. These factors create a cycle in which models achieve high accuracy on narrow benchmarks but fail to transfer, reinforcing reliance on familiar datasets.

Third, interpretability continues to be treated as an ancillary feature rather than a core design principle. Although 71.7% (66/92) of studies report some form of built-in interpretability, this high proportion is largely driven by inherently interpretable architectures (eg, attention mechanisms or feature importance rankings) rather than deliberate explainability design for clinical end users. Only 5.4% (5/92) used dedicated post hoc explanation methods such as SHAP [61] or Grad-CAM [84]. The underlying cause is a disconnect between the ML research community’s notion of interpretability (model-centric) and what surgeons need (decision-centric): actionable, real-time justifications embedded in clinical workflows rather than offline feature attribution maps.

Fourth, adaptivity to individual users is largely absent. Our analysis reveals that 91.3% (84/92) of studies use static models with no user-specific adjustment, and only 6.5% (6/92) demonstrate any form of adaptive behavior. This gap stems from a data scarcity problem at the individual level: adapting to a specific surgeon requires sufficient per-user data, which is rarely available in current datasets. Furthermore, techniques such as online learning and meta-learning that could enable few-shot personalization remain largely unexplored in the surgical domain, partly due to concerns about catastrophic forgetting and the safety implications of models that update during procedures.

Several technical limitations also arise from the nature of the data itself. Vision-based datasets, while attractive due to their ease of collection and minimal interference with the surgical workflow, present known challenges. Fukuta et al [94] reported issues such as key point occlusion, weak contrast on metallic tools, background variation, and motion blur—all of which undermine pose estimation performance, especially when moving from simulators to real-world settings. These issues are often masked in standardized setups but remain critical for deployment.

Finally, the near-total absence of system-level autonomy further underscores the field’s distance from HAT. Our mapping to the Yang et al [6] autonomy framework reveals that 96.7% (89/92) of studies operate at level 0—providing no autonomous functionality beyond classification output—while only 3 studies reach level 1 with basic assistive capabilities. No study in our corpus demonstrates level 2 or higher autonomy. This indicates that current research remains firmly in the assessment paradigm, with the transition to collaborative teaming requiring not only better models but fundamentally different system architectures that integrate perception, reasoning, and action within the surgical workflow.

These limitations point not only to the current bottlenecks but also to concrete directions where further development is needed. The following section outlines key research priorities that can support the transition toward HAT-readiness in surgery.

### Research Opportunities for HAT Readiness

The limitations identified in the preceding section point to 6 concrete research priorities that can accelerate the transition from skill assessment toward functional HAT in surgery. These priorities represent the authors’ forward-looking recommendations, informed by the gaps identified in this review, rather than direct findings from the 92 included studies.

First, addressing the data bottleneck that confines 77.2% (71/92) of studies to TRL 3 (“Limitations and Research Gaps in Current Literature” section) will require coordinated, multicenter dataset initiatives. Federated learning offers a promising pathway, enabling institutions to collaboratively train models on distributed clinical data without sharing sensitive recordings, thereby sidestepping many privacy and ethics barriers. In parallel, domain adaptation and transfer learning techniques—such as adversarial domain alignment and curriculum-based fine-tuning—should be systematically investigated to bridge the simulation-to-clinical gap. Synthetic data augmentation through procedurally generated surgical scenes or physics-based instrument simulations could further expand training distributions without additional clinical data collection.

Second, the near-absence of user adaptivity (84/92, 91.3% of studies use static models; “Limitations and Research Gaps in Current Literature” section) demands dedicated investigation into personalization mechanisms. Concrete approaches include (1) meta-learning frameworks (eg, model-agnostic meta-learning) that learn transferable initialization parameters, enabling rapid few-shot adaptation to a new surgeon from minimal calibration trials; (2) online continual learning with replay buffers to update models incrementally as per-user data accumulates, while mitigating catastrophic forgetting through elastic weight consolidation or similar regularization; (3) hierarchical Bayesian models that maintain population-level priors while estimating surgeon-specific parameters; and (4) user-calibration protocols embedded in preoperative warm-up routines, where a short standardized task establishes the individual baseline against which intraoperative performance is measured. The associated challenges—data scarcity at the individual level, safety validation of adaptive models, and the computational overhead of real-time updates—represent important open problems that the field has yet to systematically address.

Third, closing the interpretability gap between model-centric and decision-centric explainability (“Limitations and Research Gaps in Current Literature” section) requires a design-level shift. Rather than appending post hoc explanation tools to existing architectures, future systems should embed interpretability through inherently transparent modules—such as concept bottleneck layers that map internal representations to clinically meaningful features (eg, tissue tension, instrument angle, or proximity to critical structures). Uncertainty quantification via calibrated confidence estimates or conformal prediction can enable systems to communicate when they are unsure and defer to the surgeon, a prerequisite for trustworthy teaming. Co-design with surgical teams through participatory design studies will be essential to ensure that explanations are not only technically faithful but also operationally useful within the time constraints and cognitive demands of the operating room.

Fourth, incorporating patient-specific context represents a key frontier for moving from generic assessment to individualized decision augmentation. Future models should integrate preoperative imaging, anatomical variation maps, and pathology-specific risk profiles as conditioning inputs, enabling the system to anticipate procedure-specific challenges and adjust its guidance accordingly. Predictive modeling of procedural trajectories—forecasting likely next steps and potential complications based on the current surgical state—would represent a qualitative leap from retrospective assessment toward anticipatory decision support aligned with HAT requirements.

Fifth, the fragmented benchmarking ecosystem identified in this review (“Limitations and Research Gaps in Current Literature” section; RQ3) calls for community-level standardization. Specific actions include developing open, multicenter benchmark suites that span diverse procedures, skill levels, and clinical environments; adopting harmonized evaluation protocols that specify validation strategy (eg, LOUO), reporting metrics, and statistical testing; and establishing shared annotation guidelines to reduce interinstitutional labeling drift. Organizations such as the Medical Image Computing and Computer Assisted Intervention community and surgical societies are well positioned to coordinate these efforts, following precedents set by challenges such as EndoVis and Cholec80.

Finally, advancing from state recognition to procedural policy learning is essential for decision support. While current AI can identify what gesture is occurring, the critical gap is the inability to recommend what should happen next. This requires learning the underlying policy or grammar of a surgical task by abstracting low-level gestures into structured, step-based workflow representations. Sequential decision-making frameworks—including model-based reinforcement learning, inverse reinforcement learning from expert demonstrations, and probabilistic graphical models over gesture sequences—provide the architectural foundation for systems capable of modeling optimal procedural paths and providing guidance tailored to a surgeon’s proficiency. This direction represents perhaps the most ambitious yet transformative step toward genuine human-autonomy collaboration in surgery.

Addressing these priorities in a coordinated manner offers the opportunity to advance from isolated AI tools toward intelligent, adaptive surgical teammates capable of supporting decision-making, enhancing training, and improving patient outcomes.

### Translating AI Skill Assessment to HAT

The transition from AI-driven surgical skill assessment to true HAT represents not only a technical challenge but also a sociotechnical one. Effective deployment of HAT systems in clinical practice demands a holistic approach that integrates technological capabilities with human factors, workflow dynamics, regulatory requirements, and organizational considerations. Our finding that 84.8% (78/92) of studies discuss clinical integration only briefly (Table 1) underscores how underexplored these translational dimensions remain.

A primary barrier is the requirement for real-time performance under strict safety constraints. Intraoperative AI systems must deliver inference within milliseconds to avoid disrupting surgical flow, yet must simultaneously maintain high reliability—false or delayed feedback during a critical procedural step could compromise patient safety. This creates an engineering tension between model complexity and latency that few current studies address: only 25% (23/92) of reviewed studies demonstrated real-time capability, and most of these were validated in controlled rather than live operating room conditions. Hardware constraints further complicate deployment, as operating rooms may lack the GPU infrastructure required for computationally intensive deep learning inference, necessitating either model compression techniques (eg, pruning, quantization, or knowledge distillation) or edge computing architectures optimized for surgical environments.

Seamless integration into existing surgical workflows represents another critical challenge. AI systems must be designed to complement, rather than disrupt, established practices. This requires careful consideration of how feedback is delivered (eg, visual overlays, auditory cues, or haptic signals), when it is delivered (avoiding information overload during high-cognitive-demand phases), and how autonomy levels are modulated based on context and user preferences. Human-centered design principles and codevelopment with surgical teams are essential to ensure usability, trust, and adoption. Importantly, the mode of feedback must be tailored to the surgical context: a real-time warning during a high-risk dissection step requires a fundamentally different interface design than a postoperative performance summary for training purposes.

Surgeon acceptance and trust represent perhaps the most underappreciated translational barrier. Surgeons are trained to exercise autonomous clinical judgment, and introducing an AI system that offers guidance or correction during procedures raises concerns about professional autonomy, overreliance on technology, and the potential for skill degradation over time. Building trust requires not only technical reliability but also transparency in system behavior: AI must communicate its confidence levels, provide clear justifications for its recommendations, and defer to human expertise when faced with uncertainty. Participatory design—involving surgeons as codevelopers rather than end users—and graduated exposure through simulation-based familiarization can help bridge the acceptance gap. Evidence from adjacent domains such as aviation crew resource management suggests that teaming is most effective when both human and autonomous agents have clearly defined, mutually understood roles.

Regulatory and medico-legal considerations present additional hurdles that the reviewed literature largely overlooks. AI systems that influence intraoperative decisions will require regulatory clearance through pathways such as the US Food and Drug Administration’s Software as a Medical Device framework or the EU Medical Device Regulation, both of which demand rigorous clinical validation, risk classification, and postmarket surveillance. The question of liability is equally unresolved: if an AI-guided intervention leads to an adverse outcome, the allocation of responsibility among the surgeon, the institution, and the system developer remains legally ambiguous in most jurisdictions. These uncertainties create institutional risk aversion that may slow adoption even when technical readiness is achieved. Proactive engagement with regulatory bodies and the development of domain-specific guidelines for surgical AI will be necessary to establish clear pathways from prototype to clinical deployment.

Finally, iterative evaluation in real-world settings is necessary to refine HAT systems continuously. Pilot deployments, multi-institutional collaborations, and longitudinal studies will help identify practical barriers, optimize system performance, and ensure that AI teammates contribute meaningfully to surgical outcomes and team dynamics. Such evaluations should assess not only technical performance metrics but also human factors outcomes—including cognitive workload, situation awareness, team communication patterns, and long-term skill development trajectories—to ensure that AI integration enhances rather than undermines surgical team performance.

By addressing these translational challenges, AI systems can evolve from passive assessment tools into active, adaptive teammates that enhance surgical decision-making, training, and patient care.

### Conclusion

This scoping review successfully examined the landscape of AI-based methods for surgical skill assessment, focusing on their potential to enable HAT. Our synthesis provides clear answers to the 3 RQs that guided this review:

Regarding real-time HAT adaptation and guidance (RQ1), we conclude that current AI systems require significant adaptation to transition from post hoc assessment to real-time teaming. This demands that future HAT models incorporate adaptive, personalized feedback mechanisms and address critical challenges in ecological validity and interpretability. Necessary adaptations include embedding uncertainty communication and ensuring seamless alignment of AI outputs with existing surgical workflows.

Regarding ML methods and multimodal data enhancement (RQ2), significant technical progress has been made, with deep learning becoming the dominant methodology (n=55, 59.8% of studies) for movement classification. Multimodal data fusion (combining video, kinematics, and biosignals), enhanced by architectural strategies such as attention mechanisms and GCNs, is essential for integrating these diverse data streams to improve the robustness and reliability of surgical movement classification for real-time feedback.

Regarding benchmarks and expert evaluations (RQ3), while standardized rubrics such as OSATS and GRS serve as the ground truth for expert evaluation, the AI landscape is characterized by inconsistent benchmarking protocols. The prevalent reliance on small, simulated datasets (eg, JIGSAWS) limits the ability to generalize results and accurately compare AI performance with established expert skill rankings in diverse clinical environments.

Looking ahead, advancing HAT in surgery requires not only technical innovation but also a foundation built on human-centered design, strict adherence to regulatory considerations, and iterative validation in real-world clinical settings. By addressing these multifaceted challenges, AI systems can evolve into intelligent surgical teammates that enhance decision-making, support skill development, and ultimately contribute to improved patient care.

## Appendix

Multimedia Appendix 1 PRISMA-ScR checklist.

Multimedia Appendix 2 Database search strategies.

Multimedia Appendix 3 Full per-study assessment.

## Abbreviations

ANN: artificial neural network
AR: augmented reality
AUC: area under the curve
Bi-LSTM: bidirectional long short-term memory
BoF: bag of features
Cholec80: Cholecystectomy 80
CNN: convolutional neural network
ConvLSTM: convolutional long short-term memory
ESSQS: Endoscopic Surgical Skill Qualification System
GCN: graph convolutional network
GOALS: Global Objective Assessment of Laparoscopic Skills
Grad-CAM: gradient-weighted class activation mapping
GRS: Global Rating Scale
HAT: human-autonomy teaming
HMM: hidden Markov model
I3D: Inflated 3D ConvNet
JIGSAWS: Johns Hopkins University and Intuitive Surgical Inc Gesture and Skill Assessment Working Set
LOUO: leave-one-user-out
LSTM: long short-term memory
MAE: mean absolute error
ML: machine learning
MT-ViT: multitask vision transformer
OSATS: Objective Structured Assessment of Technical Skills
PRISMA: Preferred Reporting Items for Systematic Reviews and Meta-Analyses
PRISMA-ScR: Preferred Reporting Items for Systematic Reviews and Meta-Analyses extension for Scoping Reviews
RNN: recurrent neural network
RQ: research question
SHAP: Shapley additive explanations
SVM: support vector machine
TCN: temporal convolutional network
TRL: technology readiness level
U-Net: U-shaped convolutional network
ViT: vision transformer
VR: virtual reality
YOLO: You Only Look Once
