# Supplementary material for: Machine Learning for Human-Autonomy Teaming in Surgical Skill Assessment: Scoping Review
Source: JMIR AI. 2026 Oct 2;5:e94109. doi: 10.2196/94109 (PMC13675682; doi:10.2196/94109)
Supplement: Supplementary file 2 [file ai_v5i1e94109_app2.docx]

# Multimedia Appendix 1: Database Search Strategies

The following search strategies were used to identify studies for this scoping review. All searches were conducted on July 17, 2025, covering publications from January 1, 2019, to July 17, 2025. The search combined terms related to surgical skill assessment and AI/ML methods. Quotation marks were used around multi-word phrases (e.g., "surgical skill assessment", "gesture recognition surgery") to ensure exact phrase matching in all three databases. No additional filters (e.g., language, document type) were applied beyond the publication date range. In addition, 3 relevant records were identified through manual searching of reference lists and related literature.

## Database 1: Web of Science

**Date searched:** July 17, 2025

**Date range:** January 1, 2019, to July 17, 2025

**Filters applied:** Publication years 2019–2025

**Results:** 552

**Search query:**

ALL=(("surgical movement classification" OR "surgical skill assessment" OR "surgical training AI" OR "gesture recognition surgery" OR "kinematic analysis surgery" OR "surgical education") OR ("AI-assisted decision making" OR "human-autonomy teaming in surgery" OR "real-time surgical feedback" OR "personalized surgical guidance")) AND (ALL=("Artificial Intelligence" OR "Machine Learning" OR "Deep Learning")) AND PY=(2025 OR 2024 OR 2023 OR 2022 OR 2021 OR 2020 OR 2019)

## Database 2: PubMed

**Date searched:** July 17, 2025

**Date range:** January 1, 2019, to July 17, 2025

**Filters applied:** Publication date 2019–2025

**Results:** 391

**Search query:**

(("surgical movement classification"[All Fields] OR "surgical skill assessment"[All Fields] OR "surgical training AI"[All Fields] OR "gesture recognition surgery"[All Fields] OR "kinematic analysis surgery"[All Fields] OR "surgical education"[All Fields]) OR ("AI-assisted decision making"[All Fields] OR "human-autonomy teaming in surgery"[All Fields] OR "real-time surgical feedback"[All Fields] OR "personalized surgical guidance"[All Fields])) AND ("Artificial Intelligence"[All Fields] OR "Machine Learning"[All Fields] OR "Deep Learning"[All Fields]) AND (2019:2025[pdat])

## Database 3: IEEE Xplore

**Date searched:** July 17, 2025

**Date range:** January 1, 2019, to July 17, 2025

**Filters applied:** Publication years 2019–2025

**Results:** 193

**Search query:**

("surgical movement classification" OR "surgical skill assessment" OR "surgical training AI" OR "gesture recognition surgery" OR "kinematic analysis surgery" OR "surgical education") OR ("AI-assisted decision making" OR "human-autonomy teaming in surgery" OR "real-time surgical feedback" OR "personalized surgical guidance") AND ("Artificial Intelligence" OR "Machine Learning" OR "Deep Learning") AND (Publication Year: 2019-2025)

## Summary

Records from Web of Science: 552

Records from PubMed: 391

Records from IEEE Xplore: 193

Records from other sources (manual search): 3

**Total records identified: 1,139**

Duplicates removed: 633

**Records after deduplication: 506**
