# Supplementary material for: Machine Learning for Human-Autonomy Teaming in Surgical Skill Assessment: Scoping Review
Source: JMIR AI. 2026 Oct 2;5:e94109. doi: 10.2196/94109 (PMC13675682; doi:10.2196/94109)
Supplement: Supplementary file 3 [file ai_v5i1e94109_app3.docx]

Table S1. Technology readiness and translational characteristics of included studies $(n=92)$. Studies are sorted by TRL (descending), then alphabetically by first author.

| Reference | First Author (Year) | TRL | Yang | RT^a^ | Interp.^b^ | Adapt.^c^ | Val. Env.^d^ | Ext. Val.^e^ | Clin.^f^ |
| --- | --- | --- | --- | --- | --- | --- | --- | --- | --- |
| [4] | Zuluaga (2024) | 7 | 1 | RT | None | None | Clin | CS | Det |
| [91] | Ryu (2025) | 5 | 0 | RT | BI | None | Clin | No | Det |
| [103] | Abdelaal (2022) | 4 | 1 | RT | BI | Adpt | Sim | No | Brf |
| [11] | Alkadri (2024) | 4 | 0 | Off | PH | SS | Mix | CD | Brf |
| [100] | Cruz (2025) | 4 | 0 | NRT | BI | None | Lab | CS | Det |
| [36] | Ebina (2022) | 4 | 0 | Off | BI | None | Lab | CT | Brf |
| [37] | Fathabadi (2022) | 4 | 0 | NRT | BI | None | Lab | No | Brf |
| [90] | Fathabadi (2023) | 4 | 0 | NRT | BI | None | Lab | No | Brf |
| [50] | Kasa (2022) | 4 | 0 | Off | None | None | Lab | No | Brf |
| [89] | Kowalewski (2019) | 4 | 0 | Off | BI | None | Lab | CT | Brf |
| [30] | Long (2022) | 4 | 1 | RT | BI | Adpt | Lab | No | Brf |
| [3] | Natheir (2023) | 4 | 0 | Off | PH | None | Sim | No | Brf |
| [16] | Nwosu (2025) | 4 | 0 | RT | BI | None | Lab | No | Brf |
| [25] | Ryder (2024) | 4 | 0 | Off | None | None | Sim | CS | Brf |
| [53] | Shayan (2025) | 4 | 0 | Off | BI | None | Lab | CT | Brf |
| [70] | Singh (2024) | 4 | 0 | Off | BI | None | Lab | No | Brf |
| [88] | Singh (2024) | 4 | 0 | Off | BI | None | Lab | CT | Brf |
| [28] | Subedi (2022) | 4 | 0 | Off | None | None | Sim | No | Brf |
| [80] | Tonetti (2024) | 4 | 0 | Off | None | None | Sim | CD | Brf |
| [104] | Yasui (2025) | 4 | 0 | NRT | BI | None | Lab | No | Brf |
| [83] | Yibulayimu (2022) | 4 | 0 | RT | PH | None | Mix | CD | Det |
| [14] | Agarwal (2022) | 3 | 0 | Off | None | None | Lab | No | Brf |
| [32] | Ahmadi (2021) | 3 | 0 | RT | PH | None | Clin | CD | Brf |
| [48] | Anastasiou (2023) | 3 | 0 | Off | BI | None | Lab | CT | Brf |
| [33] | Anh (2020) | 3 | 0 | NRT | None | None | Sim | No | Brf |
| [99] | Bhatia (2023) | 3 | 0 | RT | BI | None | Lab | No | Brf |
| [69] | Bkheet (2023) | 3 | 0 | RT | BI | None | Lab | CT | Brf |
| [84] | Chen (2024) | 3 | 0 | Off | BI | None | Clin | CS | Det |
| [27] | Chen (2024) | 3 | 0 | Off | BI | Adpt | Mix | CD | Brf |
| [63] | Elek (2022) | 3 | 0 | NRT | BI | None | Lab | CD | Brf |
| [57] | Erlich-Feingold (2025) | 3 | 0 | Off | None | None | Lab | No | Brf |
| [72] | Fathollahi (2022) | 3 | 0 | Off | None | None | Clin | No | Brf |
| [38] | Fawaz (2019) | 3 | 0 | Off | BI | None | Sim | No | Brf |
| [35] | Fawaz (2019) | 3 | 0 | Off | BI | None | Lab | CT | Brf |
| [64] | Fukuta (2025) | 3 | 0 | NRT | BI | None | Lab | CT | Brf |
| [34] | Funke (2019) | 3 | 0 | Off | None | None | Lab | CT | Brf |
| [58] | Gao (2024) | 3 | 0 | NRT | BI | None | Lab | CS | Brf |
| [41] | Gorantla (2019) | 3 | 0 | NRT | BI | None | Lab | No | Brf |
| [56] | Guo (2024) | 3 | 0 | NRT | BI | None | Mix | CD | Brf |
| [40] | Hasani (2021) | 3 | 0 | Off | BI | None | Lab | CT | Brf |
| [78] | Hoffmann (2024) | 3 | 0 | Off | None | None | Lab | No | Brf |
| [77] | Igaki (2023) | 3 | 0 | Off | BI | None | Clin | CS | Brf |
| [42] | Iranfar (2023) | 3 | 0 | Off | BI | SS | Sim | No | Brf |
| [105] | Iranfar (2025) | 3 | 0 | Off | BI | None | Sim | No | Brf |
| [44] | Jian (2020) | 3 | 0 | Off | BI | None | Lab | CT | Brf |
| [47] | Khalil (2025) | 3 | 0 | Off | BI | None | Lab | CD | Brf |
| [93] | Khan (2024) | 3 | 0 | RT | BI | None | Clin | No | Det |
| [79] | Kim (2019) | 3 | 0 | Off | BI | None | Clin | CT | Brf |
| [15] | Kitaguchi (2020) | 3 | 0 | Off | None | None | Clin | CS | Det |
| [76] | Komatsu (2024) | 3 | 0 | RT | BI | None | Clin | CS | Det |
| [26] | Kumar (2022) | 3 | 0 | NRT | None | None | Clin | CD | Brf |
| [61] | Lajkó (2021) | 3 | 0 | Off | BI | None | Lab | CT | Brf |
| [86] | Lavanchy (2021) | 3 | 0 | Off | BI | None | Clin | No | Brf |
| [101] | Ledwos (2022) | 3 | 0 | Off | BI | None | Sim | No | Brf |
| [67] | Lee (2020) | 3 | 0 | NRT | BI | None | Mix | CT | Det |
| [94] | Lee (2025) | 3 | 0 | RT | BI | None | Clin | No | Det |
| [62] | Liu (2019) | 3 | 0 | Off | BI | None | Clin | No | Brf |
| [39] | Liu (2021) | 3 | 0 | Off | BI | None | Mix | CD | Brf |
| [75] | Liu (2022) | 3 | 0 | RT | BI | None | Mix | CD | Brf |
| [24] | Liu (2023) | 3 | 0 | RT | BI | None | Mix | CD | Brf |
| [52] | Long (2021) | 3 | 0 | RT | BI | None | Mix | CS | Brf |
| [6] | Luongo (2021) | 3 | 0 | Off | None | None | Clin | No | Brf |
| [92] | Madani (2022) | 3 | 0 | RT | BI | None | Clin | CS | Det |
| [45] | Menegozzo (2019) | 3 | 0 | RT | None | None | Mix | CD | Brf |
| [81] | Ming (2020) | 3 | 0 | Off | BI | None | Lab | CT | Brf |
| [71] | Ming (2021) | 3 | 0 | Off | BI | None | Lab | CT | Brf |
| [102] | Nakajima (2024) | 3 | 0 | RT | BI | None | Clin | CS | Det |
| [43] | Nakajima (2025) | 3 | 0 | Off | BI | None | Clin | CS | Brf |
| [82] | Pan (2023) | 3 | 0 | NRT | BI | None | Lab | CT | Brf |
| [55] | Saricilar (2023) | 3 | 0 | Off | None | None | Sim | No | Det |
| [95] | Sato (2025) | 3 | 0 | Off | BI | None | Clin | CS | Brf |
| [29] | Shafiei (2021) | 3 | 0 | Off | BI | None | Clin | CT | Brf |
| [51] | Shafiei (2024) | 3 | 0 | Off | BI | None | Lab | CT | Brf |
| [97] | Shao (2024) | 3 | 0 | RT | BI | None | Mix | CD | Brf |
| [74] | Smith (2022) | 3 | 0 | Off | None | None | Sim | CT | Brf |
| [13] | Soleymani (2021) | 3 | 0 | Off | None | None | Lab | CT | Brf |
| [54] | Soleymani (2024) | 3 | 0 | Off | BI | Adpt | Mix | CD | Brf |
| [66] | Takács (2021) | 3 | 0 | NRT | BI | Adpt | Lab | CT | Brf |
| [85] | Takeuchi (2022) | 3 | 0 | RT | BI | None | Clin | CT | Brf |
| [65] | Tang (2024) | 3 | 0 | Off | BI | None | Lab | No | Brf |
| [31] | Tanin (2024) | 3 | 0 | NRT | None | None | Clin | No | Brf |
| [96] | van Amsterdam (2020) | 3 | 0 | RT | BI | None | Lab | No | Brf |
| [49] | van Amsterdam (2022) | 3 | 0 | RT | BI | None | Mix | CD | Brf |
| [17] | Wang (2023) | 3 | 0 | Off | BI | Adpt | Sim | CD | Brf |
| [73] | Yang (2024) | 3 | 0 | NRT | BI | None | Clin | CD | Brf |
| [98] | Yanik (2023) | 3 | 0 | Off | PH | None | Lab | CD | Brf |
| [46] | Yanik (2024) | 3 | 0 | Off | BI | None | Lab | No | Brf |
| [60] | Yeh (2025) | 3 | 0 | Off | BI | None | Clin | CS | Brf |
| [59] | Yen (2025) | 3 | 0 | Off | BI | None | Clin | No | Brf |
| [87] | You (2024) | 3 | 0 | RT | None | None | Clin | No | Det |
| [10] | Zhang (2024) | 3 | 0 | Off | None | None | Sim | No | Brf |
| [68] | Zhou (2022) | 3 | 0 | Off | BI | None | Lab | CT | Brf |
| ^a^RT = Real-Time Capability: Off = Offline, NRT = Near-real-time, RT = Real-time. ^b^Interp. = Interpretability: BI = Built-in, PH = Post-hoc. ^c^Adapt. = Adaptivity: SS = Skill-stratified, Adpt = Adaptive. ^d^Val. Env. = Validation Environment: Sim = Simulation only, Lab = Lab/cadaver, Clin = Clinical, Mix = Mixed. ^e^Ext. Val. = External Validation: CT = Cross-task, CD = Cross-dataset, CS = Cross-site. ^f^Clin. = Clinical Integration Discussed: Brf = Brief, Det = Detailed. TRL = NASA Technology Readiness Level (1–9); Yang = Yang Autonomy Level (0–5). | | | | | | | | | |
